# The spatial extent and the dispersal strategy of species shape the occupancy frequency distribution of stream insect assemblages

**DOI:** 10.1002/ece3.11663

**Published:** 2024-07-10

**Authors:** I. Szivák, Z. Csabai, D. Schmera, A. Móra

**Affiliations:** ^1^ HUN‐REN Balaton Limnological Research Institute Tihany Hungary; ^2^ Faculty of Sciences, Department of Hydrobiology University of Pécs Pécs Hungary

**Keywords:** dispersal processes, dispersal strategy of species, niche processes, occupancy frequency distribution, spatial extent of sampling, stream insects

## Abstract

Several theoretical models have been proposed as the underlying mechanisms behind occupancy frequency distribution (OFD) patterns. For instance, the metapopulation dynamic model predicts bimodal OFD pattern indicating the dominance of dispersal processes in structuring the assemblages, while the niche‐based model predicts unimodal right‐skewed OFD pattern, and thus assemblages are driven mostly by niche processes. However, it is well known that the observed OFD pattern reflects the interplay of several other factors (e.g. habitat heterogeneity, species specificity and sampling protocol parameters). It follows that the individual contribution of each factor to the OFD pattern is rather complicated to explore. Our main objective was to examine the role of the spatial extent of the sampling and the dispersal strategies of species in shaping OFD pattern. For this, we collected samples of stream insect assemblages inhabiting near‐natural streams in the Pannon Ecoregion. We formed groups of species representing contrasting dispersal strategies (referred to as dispersal groups). Applying a computer program algorithm, we produced samples with different spatial extent. We found that with increasing spatial extent, the OFD pattern changed from bimodal to unimodal for active dispersers. Insect groups with different dispersal strategies differed in the strength of support for OFD patterns within all spatial extent. Furthermore, the strength of support for OFD patterns varied across dispersal groups differently as the spatial extent increased. Our results reflected underlying changes in mechanisms structuring assemblages along an increasing spatial extent. We also assumed that the stream insect dispersal strategy influences the relative role of dispersal and niche processes particularly as spatial extent increases from stream reaches to the extent of adjacent valleys. We could define spatial extents and dispersal strategies within which unique metacommunity processes could underlie the organisation of assemblages.

## Graphical abstract

We examined the role of spatial extent of sampling and dispersal strategies of stream insect species in shaping occupancy frequency distribution (OFD) in near‐natural streams in the Pannon Ecoregion. With increasing spatial extent, the OFD pattern changed from bimodal to unimodal for active dispersers. Insect groups with different dispersal strategies differed in the strength of support for OFD patterns within all spatial extent. We could define spatial extents and dispersal strategies within which unique metacommunity processes could underlie the organisation of assemblages.

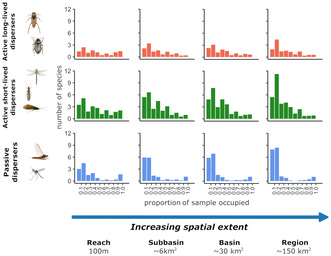

## 1 INTRODUCTION

Patterns of species occupancy frequency distributions (OFDs) are widely studied by community ecologists because they support the inference on the underlying mechanisms driving communities (McGeoch & Gaston, 2002). OFDs are histograms that represent the number of species that occur at a given proportion of sites, therefore providing information on the relative dominance of rare and common species in ecological communities. It can take multiple shapes, but three of them received particular attention: right‐skewed unimodal, bimodal and uniform (called also random) OFD patterns (McGeoch & Gaston, 2002; Tokeshi, 1992). The first two patterns indicate well‐structured communities, organised by various local and regional filters. The unimodal right‐skewed OFD is characterised by a rare species mode (referred as the satellite mode), while bimodal OFD shows an additional common species mode (referred as the core mode).

Several theoretical models have been proposed as underlying processes behind OFD patterns (see reviews by Hui, 2012; Jenkins, 2011; McGeoch & Gaston, 2002; Tokeshi, 1992). However, two mutually non‐exclusive models received particular attention. The metapopulation dynamic model (model 1) suggests that the occupancy dynamics is balanced by local colonisation of newly arriving species and by the local extinction of already existing species (Hanski, 1982). This model puts emphasis on dispersal processes (Brown et al., 2011). The niche‐based model (model 2) emphasises the idea that the abundance and occupancy of species are limited by the combination of physical and biotic factors (Brown et al., 2011; Brown, 1984). The metapopulation dynamic predicts a bimodal OFD pattern (Hanski, 1982), while the niche‐based model predicts a unimodal right‐skewed OFD pattern (Brown, 1984).

Despite its apparent simplicity, the OFD pattern could be influenced by several biological and environmental factors such as species specificity (e.g. dispersal potential, range size, niche requirement), community structure (e.g. species number and nestedness rate), metacommunity succession, environmental condition (e.g. habitat heterogeneity), geographical location and disturbance regimes (e.g. Hui, 2012; Jenkins, 2011; McGeoch & Gaston, 2002). All these factors could modify the occupancy of the species through an increasing proportion of satellite or core species. Obviously, the observed OFD pattern reflects the interplay of these factors and is also influenced by sampling parameters (Heatherly et al., 2007; see Table 5 in Kammer & Vonlanthen, 2009). It follows that the individual contribution of each factor to OFD pattern is rather complicated to explore (Gafta et al., 2021). For instance, bimodality is associated with sampling within similar habitats located in a small, environmentally homogeneous area where all species could reach all potentially available sites (Hui, 2012). Some authors argued that bimodality might arise only as a consequence of the statistical properties of species occupancy probability distributions (Hui & McGeoch, 2007) or a pure artefact of the sampling protocol: for example, when sample unit size is relatively large compared to the sample extent (Kammer & Vonlanthen, 2009), and when rare species are less likely to be observed or sampled (Nee et al., 1991; van Rensburg et al., 2000). The general interpretation of OFD patterns is thus challenging because the shape of OFDs depends on the scale of the sampling (Collins & Glenn, 1997; Hui, 2012; Jenkins, 2011; Kammer & Vonlanthen, 2009).

The spatial extent dependence of OFDs (called also as distance scaling principle) is among the most investigated aspects of OFD studies (Collins & Glenn, 1997; Heatherly et al., 2007; Jenkins, 2011; Kammer & Vonlanthen, 2009; van Rensburg et al., 2000). It predicts that with an increasing spatial extent of the sampling (referred as spatial extent), the proportion of satellite species will increase, while the proportion of core species will decrease. Consequently, the OFD pattern shifts from bimodal to unimodal (McGeoch & Gaston, 2002). In terrestrial ecosystems, several empirical evidences support the crucial role of spatial extent: studies on grasshoppers, small mammals, plants, birds and dung beetles already indicate the existence of the phenomenon (Collins & Glenn, 1997; Suhonen & Jokimäki, 2019; van Rensburg et al., 2000). Regarding freshwater ecosystems, however, the number of case studies is limited and yielded contradictory results: in some cases, the shift was confirmed (insects: Heatherly et al., 2007), while in others, the OFD pattern remained unimodal independently from the spatial extent (insects: Heino, 2015; macrophytes: Suhonen, 2021). Thus, in freshwater ecosystems, the dependency of OFD patterns on spatial extent is understudied, and the findings are ambiguous.

Beside spatial extent, the dispersal of species can also influence OFD patterns (the phenomenon called also the organismal scaling principle: Collins & Glenn, 1997; Suhonen et al., 2022). Organismal scaling principle predicts that species with good dispersal abilities can colonise many sites and thus increase the number of core species (contributing to the bimodal OFD pattern), while species with limited dispersal abilities can colonise only a restricted number of sites, and thus increase the number of satellite species (contributing to the unimodal OFD, see Collins & Glenn, 1997). Several studies underpinned the relationship between the dispersal of species and OFD patterns (e.g. Collins & Glenn, 1997; Heino, 2015; Korkeamäki et al., 2018; van Rensburg et al., 2000). Regarding freshwater insect assemblages, however, the support of this hypothesis seems to be weak if dispersal groups are defined based on taxonomy (e.g. Collins & Glenn, 1997; Mehranvar & Jackson, 2001; Suhonen et al., 2022; van Rensburg et al., 2000). Similarly, the hypothesis did not receive support when dispersal was characterised by individual traits (e.g. Heino, 2015; Korkeamäki et al., 2018), probably because using one trait is not sufficiently strong proxy to describe the dispersal ability and the colonisation success of species. It is well known that some dispersal‐related traits showed trade‐offs with each other (e.g. body size and fecundity, Schmera et al., 2022; Verberk et al., 2008, 2013; Wilkes et al., 2020). This implies that a species could have good dispersal ability even if it seems weak disperser based on one selected dispersal trait. A new and promising approach is to split communities into different functional groups based on several traits and define their dispersal strategies. Focusing on these dispersal strategies instead of individual traits could be suitable for finding more general ecological patterns (e.g. OFDs), since it could provide a more complete view of the role that dispersal plays in shaping community assembly (Sarremejane et al., 2021; Tonkin, Altermatt et al., 2018a; Verberk et al., 2008, 2010, 2013).

In the present study, we examine the effects of the spatial extent of sampling and the dispersal strategies of stream insect species on the OFD pattern of insect assemblages. For this purpose, we collected samples of freshwater insect assemblages that inhabit near‐pristine temperate streams in the Pannon Ecoregion. Relying on species traits, we formed groups of species representing contrasting dispersal strategies. Applying a computer program algorithm, we produced representative samples with different spatial extents. We expected that our study on freshwater organisms could support the spatial extent dependence of OFDs according to distance scaling principle (i.e. shifts from bimodal to unimodal pattern with increasing spatial extent), as observed in terrestrial ecosystems. We also expected that the use of combinations of dispersal traits could be suitable for revealing differences in OFD patterns among insect groups with different dispersal strategies, as it reduces many complex and diverse relationships to a few meaningful ones.

## 2 MATERIALS AND METHODS

### 2.1 Study area and sampling

**FIGURE 1:**
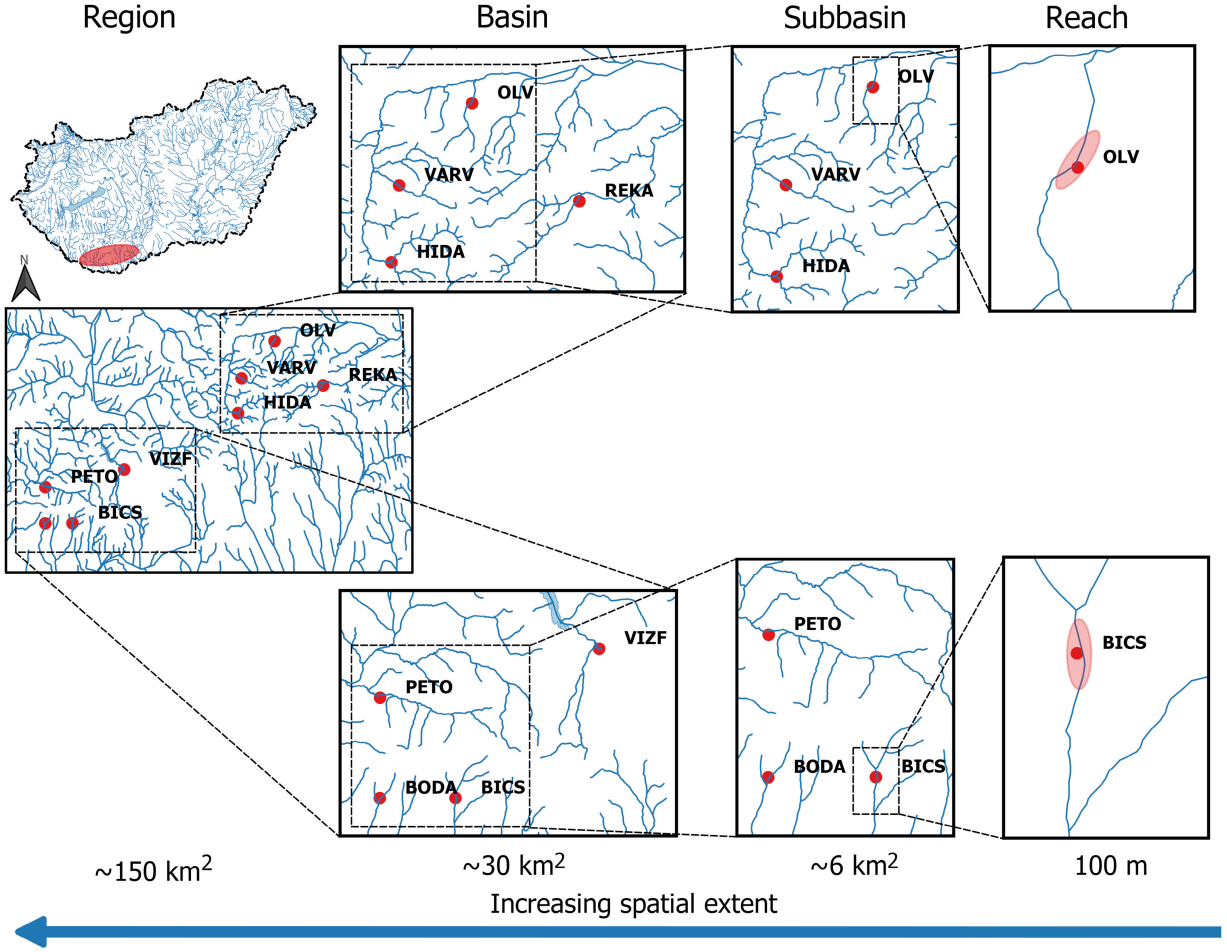
Map of study sites in the Mecsek Mountains at four different spatial extents (reach, subbasin, basin, region) along increasing spatial scale. As study sites, 100 m long stream reaches were investigated (referred to as the reach extent) in distinct second‐order stream valleys. Six of these reaches belong to two subbasins of third‐order streams with three stream reaches in each (referred to as the subbasin extent). By including an additional reach to previous three ones, we obtained a larger spatial extent covering river basin areas (referred to as the basin extent). Analysing all samples from the eight stream reaches collectively, it is referred to as the regional extent.

The survey area is located in the Mecsek Mountains (Hungary), which is one of the most southern mountain ranges in the Carpathian Basin (Figure 1). It is considerably isolated from other mountain regions and is surrounded by plains and low hilly territories. This relatively small (350 km^2^), geologically and climatically heterogeneous area is located in a biogeographic transition zone (Praeillyricum) between the Pannonian and the Mediterranean (Dinaric, Illyrian, Moesian) ecoregions (Borhidi, 2003).

The spatial location of the selected eight study sites allowed us to study stream insect assemblages at four different spatial extents along increasing spatial scale: reach (100 m), subbasin (~6 km^2^), basin (~30 km^2^) and region (~150 km^2^) (see Figure 1 and Table A1 in Appendix 1). The study sites are situated in distinct second‐order stream valleys, where 100 m long stream reaches were investigated (referred to as the reach extent). Six of these reaches belong to two subbasins of third‐order streams (Völgységi and Bükkösdi streams), with three stream reaches in each (referred to as the subbasin extent). For each subbasin, an additional reach was selected, located farther away from the existing three. By including this new reach, we obtained four reaches covering larger river basin areas: four reaches within the Danube basin and four within the Drava basin (referred to as the basin extent). Finally, when analysing all samples from the eight stream reaches collectively, it is referred to as the regional extent. As a seasonally biased sample might underestimate the number of rare species and consequently bias OFD patterns (McGeoch & Gaston, 2002), we sampled stream insects four times (May 2009, July 2009, October 2009 and March 2010). This temporal replicate sampling allowed representative assessment of stream insect assemblages of the studied reaches at annual scale (Gaston & Lawton, 1989; Tokeshi, 1992).

Before sampling, we divided each 100 m long reaches into 20 transects of similar length and drew microhabitat coverage maps. Based on these maps, we estimated the percentage coverage of microhabitat types within reaches. The kick and sweep sampling technique was used to collect stream insects using a hand net (frame width: 25 cm, mesh size 1000 μm) by disturbing an area of 0.25 × 0.25 m. We considered this as the elementary sample unit of the study. Using stratified random multihabitat sampling procedure, we collected 20 elementary sample units from the major microhabitats in proportion to their presence within reaches to gain a representative sample (AQEM protocol, Hering et al., 2003, 2004). In total, we handled 640 elementary sample units for further statistical analyses: 20 elementary sample units (0.25 × 0.25 m) per stream reach; sampling was repeated four times per year at eight stream reaches in Mecsek Mountains. Elementary sample units were kept and processed separately. Each sample unit was fully and accurately sorted in the laboratory. Insect species of eight taxonomic groups (Coleoptera, Heteroptera, Ephemeroptera, Odonata, Plecoptera, Megaloptera, Trichoptera, Diptera: Chironomidae) were included in this study. Other dipterans were identified at higher taxonomic extents such as family or infraorder (e.g. Tipulomorpha). Identification was carried out using relevant keys and descriptions (Appendix 2).

### 2.2 Characterisation of stream insect dispersal strategy

To classify taxa based on their dispersal strategy, we selected eight dispersal‐related morphological (maximum body size and female wing length), behavioural (dispersal strategy and propensity to drift) and life‐history traits (life cycle duration, adult life span, reproductive cycles per year and life‐long fecundity) (Table C1 in Appendix 3) sourced from the DISPERSE database (Sarremejane et al., 2020). Several trait categories were assigned to each trait (see Table C1 in Appendix 3). In the database, the value of trait categories was given by a fuzzy‐coding approach, specifying whether the taxon has no (0), weak (1), moderate (2) or strong (3) affinity with the given trait category. These trait affinities were standardised as proportions, ranging from 0 to 1, so that they described relative trait occurrences (Bêche et al., 2006; Sarremejane et al., 2021; Schmera et al., 2022). Two morphological traits (body size and female wing length) originally contained many trait categories (7 and 8, respectively) that could make it difficult to interpret the results. Therefore, these trait categories were merged into three newly created trait categories that contained wider size and length ranges (Table C1 in Appendix 3). We assigned the highest affinity value to the new trait categories, which occurred in the original trait categories merged into them. In the database, most of the traits of freshwater insects were coded at the genus level, but some Diptera taxa were coded at the family, subfamily or tribe levels. When taxa were identified at the family level, but data were given at the genus level, we calculated a mean value based on all genera in the higher rank taxon (Bêche et al., 2006; Sarremejane et al., 2021). As multilevel pattern analysis (see below) could not handle missing values, we substituted rarely encountered missing values by the mean value based on all genera in the same taxonomical category (e.g. family and superfamily).

We identified clusters of taxa with similar dispersal trait profiles (referred to as dispersal groups) following the method described in Sarremejane et al. (2021). Specifically, we executed hierarchical cluster analysis with Ward's method (using Gower dissimilarity metrics). Verifying the suitability for Ward's clustering method, we ensured that the Gower dissimilarity matrix met the criterion of Euclidean distance, with positive eigenvectors derived from a principal component analysis of a double‐centred dissimilarity matrix (Aspin et al., 2019; Bruno et al., 2016). We applied the elbow method to define the optimal number of clusters by minimising the total within‐cluster sum of squares (WSS). In this approach, we visually determined the optimal number of clusters by plotting WSS against the number of clusters. Based on this plot, we selected a number of clusters, where including an additional cluster did not enhance the total WSS. The optimal number of clusters could be indicated by the presence of a bend (knee) in the plot. We verified the resulted clusters by ANOSIM analysis (999 permutation). Finally, we conducted multilevel pattern analysis (a kind of indicator species analysis) to find highly indicative trait categories for each cluster (De Cáceres et al., 2010; De Cáceres & Legendre, 2009). This approach provides an index of the association strength (point‐biserial coefficient) for each trait category within the a priori defined clusters and their combinations. The degree of statistical significance of these associations was tested using permutation tests (999 permutations). Statistical analyses were performed in R ver. 4.2.2 (R Core Team, 2022) software environment using the following functions and packages: ‘hcut’ and ‘anosim’ in *vegan* (hierarchical cluster analysis and ANOSIM, Oksanen et al., 2022), ‘fviz_nbclust’ in *factoextra* (determination of optimal number of clusters, Kassambara & Mundt, 2020), and ‘multipatt’ in *indicspecies* (multilevel pattern analysis, De Cáceres & Legendre, 2009). For graphical outputs, we applied the following packages: *ggdendro* (de Vries & Ripley, 2022).

### 2.3 Re‐sampling procedure

The seasonally repeated sampling procedure, the separate handling of sample units, and the different extents of the stream habitat allowed us to produce annually and spatially representative samples with different spatial extents.

1. The reach‐extent sample was composed as follows: (1) we randomly selected one from eight stream reaches, with its complete seasonal sampling, resulting in 20 × 4 sample units. (2) From these 80 sample units, we randomly chose four, ensuring that one is selected from each season. (3) We pooled these sample units to obtain a representative sample with respect to an annual period (referred to as annually representative sample – ARS), thus one ARS represented 4 × 0.25 × 0.25 m sampling area. (4) We repeated steps 2 and 3 with replacement 16 times and obtained 16 ARSs. We considered these 16 ARSs together as a spatially representative sample (SRS). (5) We repeated steps 1–4 with replacement 10,000 times to get 10,000 reach‐extent SRSs.
2. At the subbasin extent, we randomly selected one from two subbasins consisting of three stream reaches, with its complete seasonal sampling, resulting in 20 × 4 × 3 sample units. For each stream reach we obtained five ARSs applying steps 2–4 described above for reach‐extent samples. We considered 3 × 5 ARSs together as subbasin‐extent SRS. We repeated this procedure with replacement 10,000 times to get 10,000 subbasin‐extent SRSs. In this case, we selected 15 ARSs instead of 16. This was necessary to ensure balanced sampling, meaning an equal number of ARSs from each of the three stream reaches.
3. At the basin extent, we randomly selected one from two basins consisting of four stream reaches with its complete seasonal sampling, resulting in 20 × 4 × 4 sample units. For each stream reach we obtained four ARSs applying steps 2–4 described in the first case. We considered 4 × 4 ARSs together as basin‐extent SRS. We repeated this procedure with replacement 10,000 times to get 10,000 basin‐extent SRSs.
4. At the regional extent, we randomly selected sample units from all stream reaches, resulting in 20 × 4 × 8 sample units. For eight stream reaches we obtained two ARSs. We considered 2 × 8 ARSs together as regional‐extent SRS. We repeated this procedure with replacement 10,000 times to get 10,000 regional‐extent SRSs.

We discarded all ARSs with less than 10 taxa, because a low taxonomic richness does not give a reliable OFD pattern (McGeoch & Gaston, 2002).

### 2.4 OFD patterns of stream insects

To identify different OFD patterns, we analysed empirical ranked species occupancy curves (RSOCs as in Hui, 2012; Jenkins, 2011) by regression and multimodel inference. The RSOC method is mathematically transferable from OFD (Hui, 2012; Jenkins, 2011); however, it is a more operational alternative to OFDs since it provides an objective test of different shapes, and gives parameter estimates (Gafta et al., 2021; Jenkins, 2011). Additionally, the RSOC method keeps the species identity information (see Figure 3 in Jenkins, 2011). Therefore, it has recently gained more attention in empirical studies (Gafta et al., 2021; Korkeamäki et al., 2018; Renner et al., 2020; Suhonen, 2021; Suhonen et al., 2022). At first, for each SRS, we assessed relative occupancies (*O*_*i*_) per taxa by counting the number of ARSs where the given taxa were found and dividing it by the total number of ARSs in a SRS. Second, we constructed RSOCs by sorting the relative occupancies of taxa in decreasing order and then plotting the relative occupancy of the taxa as a function of its rank (R_i_). At the third stage, we computed one linear and four nonlinear regressions of RSOCs using relative occupancy of taxa (*O*_*i*_) as the dependent variable, and rank values (*R*_*i*_) as the independent variable. Following Jenkins' (2011) procedure, nonlinear regressions were calculated using the Levenberg–Marquardt algorithm (with 999 iterations) and the means of ordinary least squares (OLS). We estimated the parameters (*y*_*0*_, *a*, *b*, *c*) of five RSOC regression models equivalent to five most common OFD patterns using the equations in Table 1 (Hui, 2012; Jenkins, 2011). Finally, we computed the Akaike information criterion score for small sample size (AICc) and the Akaike weight (*w*; Anderson et al., 2000) for each regression equation on each SRS. Akaike weights provide a relative weight of evidence for each model, thus can be considered as analogous to the probability that one given model is the best approximation among all competing models (Burnham et al., 2011; Burnham & Anderson, 2002; Garamszegi & Mundry, 2014; Johnson & Omland, 2004; Symonds & Moussalli, 2011). Therefore, we kept the Akaike weights of the regression models in each SRS and referred them as quantification of model selection uncertainty.

**TABLE 1:** Five RSOC regression models which are equivalent to most common OFD patterns and the equations for RSOC model with their initial parameters.

RSOC model	OFD pattern	Equation
Exponential concave	Unimodal satellite dominant	O_i_ = *y*_*0*_ + *a ×* exp(−*b*R_i_) with initial parameters *y0* = 0.01, *a* = 1.0, *b* = 0.01
Power exponential	Bimodal truncated OFD if *b* > 0 Unimodal truncated OFD if *b* ≤ 0	O_i_ = *a*R_i_^b^ *×* exp(−*c*R_i_) with initial parameters *y0* = 1, *a* = 0.01, *b* = 0.01
Sigmoidal symmetric	Bimodal symmetric	O_i_ = *a*/(1 + exp(−*b*R_i_ + *c*)) with initial parameters *a* = 1.0, *b* = −0.1, *c* = −1.0
Sigmoidal asymmetric	Bimodal asymmetric	O_i_ = *a*(1‐exp(−*b*R_i_^*c*^)) with initial parameters *a* = 1.0, *b* = −1.0, *c* = −1.0
Linear	Uniform or random	O_i_ = *a*R_i_ + *b*

In our study, we did not choose a single best model for each dataset based on Akaike weights, rather we applied multi‐model inference which enables formal inference from a set of models instead of one (Burnham & Anderson, 2002; Garamszegi & Mundry, 2014; Johnson & Omland, 2004; Symonds & Moussalli, 2011). This approach is adequate to use in cases where no single model is overwhelmingly supported by the data (i.e. Akaike weight < 0.9; Johnson & Omland, 2004; Symonds & Moussalli, 2011). In such cases, it would be misleading to simply select the best model and discard the others, since the selected model might not explicitly describe the empirical ecological pattern (Garamszegi & Mundry, 2014; Johnson & Omland, 2004; Symonds & Moussalli, 2011). In our study, a single RSOC model very rarely attained an Akaike weight value exceeding 0.9; therefore, we applied multi‐model inference based on all RSOC models.

The multi‐model inference also enabled us to assess the relative importance of predictor variables or a functional form representing a specific biological pattern. It can be calculated as the sum of Akaike weights across all models where the variable or the functional form of interest is present (Burnham & Anderson, 2002; Johnson & Omland, 2004; Symonds & Moussalli, 2011). Consequently, we did not specify uncertainty for individual RSOC models; instead, we identified levels of support for functional forms representing the unimodal, bimodal and random OFD patterns for each SRS. The bimodal OFD is mathematically equal to the sigmoidal RSOC indicated by the existence of an inflection point (Hui, 2012). Among the five models, the sigmoidal symmetric, sigmoidal asymmetric and power exponential model with *b* > 0 have an inflection point, thus representing the bimodal OFD pattern (Hui, 2012). The exponential concave and the power exponential model with *b* ≤ 0 represent the unimodal OFD pattern. The linear RSOC is equivalent to uniform or random OFD pattern (termed hereafter as random OFD pattern). Thus, we estimated the probability of best fit of the bimodal OFD pattern (referred as probability of bimodal OFD pattern) by summing the Akaike weights of the sigmoidal symmetric, sigmoidal asymmetric and the power exponential model with *b* > 0. The probability of best fit of the unimodal OFD pattern (referred to as probability of unimodal OFD pattern) was calculated by summing the Akaike weights of the exponential concave and the power exponential model with *b* ≤ 0.

To assess the level of the support for each OFD model within spatial extents and dispersal groups, we plotted the mean and confidence interval of Akaike weights󠄀 for 10,000 SRSs against increasing spatial scale separately for each dispersal group. We calculated evidence ratio (*ER*) derived from mean Akaike weights (*w*_*i*_ */w*_*j*_) for all OFD pattern pairs (bimodal–unimodal, random–bimodal and random–unimodal patterns) to follow how the level of support for a given OFD pattern over another changes across spatial extents and dispersal groups. Evidence ratio is beneficial in comparing the relative support for one model (e.g. bimodal OFD) compared to another (e.g. unimodal OFD), and independent of other models in the set (Burnham et al., 2011; Burnham & Anderson, 2002; Wagenmakers & Farrell, 2004). We also presented the empirical OFD histograms for each spatial extent and dispersal group to visualise our results.

To estimate the effect of sampling extent and dispersal traits on OFD patterns, we kept other variables constant (e.g. the size and the number of sample units, sampling intensity) or controlled them (e.g. habitat heterogeneity and taxa number). To investigate how the number of taxa influences the OFD patterns across different spatial extents and dispersal groups, we first calculated the number of taxa for all SRSs. Then, we conducted generalised linear mixed models (GLMMs) with beta flexible distribution family and logit function. We examined the effects of taxa number and spatial extent, as well as the influence of taxa number and dispersal group on the Akaike weights of unimodal and bimodal patterns in separate GLMM models. In the models, the Akaike weights of OFD patterns for all SRSs were used as the response variables. The number of taxa for all SRSs was considered as the continuous explanatory variable, while spatial extent, and dispersal group were included as categorical explanatory variables. Furthermore, we involved individual assemblages within spatial extents and dispersal groups (i.e. assemblages defined by dispersal groups, and assemblages in each spatial extent, respectively) as random factors.

To estimate whether habitat heterogeneity had a significant contribution to OFD patterns, we examined differences in instream microhabitat heterogeneity among eight stream reaches. We run PERMDISP analysis, and the Tukey HSD test on the coverage percentage of microhabitat types as explanatory variables and the identity of stream reaches as grouping variables. We performed this analysis for one annual dataset. We assessed the impact of microhabitat heterogeneity on taxa number using Spearman's rank correlation tests. The tests were conducted with the annual cumulative taxa number in reaches and the mean distance to the group centroid, derived from PERMDISP analysis. The mean distance values indicate the level of microhabitat heterogeneity. Statistical analyses were performed in R ver. 4.2.2 (R Core Team, 2022) software environment using the following functions and packages: ‘betadisper’ in *vegan* (PERMDISP analysis, Oksanen et al., 2022), ‘nlsLM’ in *minpack*.*lm* (nonlinear regressions of RSOCs, Elzhov et al., 2022), and ‘AICc’ and ‘Weights’ in *MuMIn* (AIC, and Akaike weights, Barton, 2022), ‘glmmTMB’ in *glmmTMB* (GLMM, Brooks et al., 2017). For graphical outputs, we applied the following packages: *ggplot2* (Wickham, 2016), *patchwork* (Pedersen, 2023), and *ggeffects* (Lüdecke, 2018).

## 3 RESULTS

### 3.1 Dispersal strategies of stream insects

**FIGURE C1:**
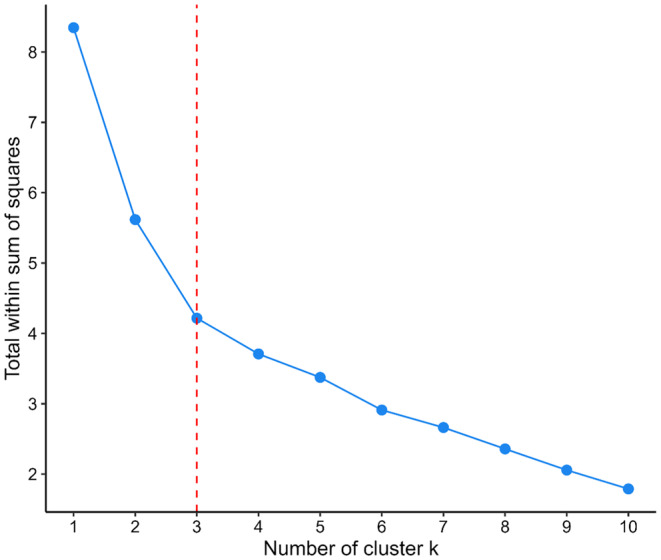
We visually determined the optimal number of clusters by plotting WSS against the number of clusters. We selected a number of clusters, where including an additional cluster does not enhance the total WSS. The optimal number of clusters could be indicated by the presence of a bend (knee) in the plot.

**FIGURE C2:**
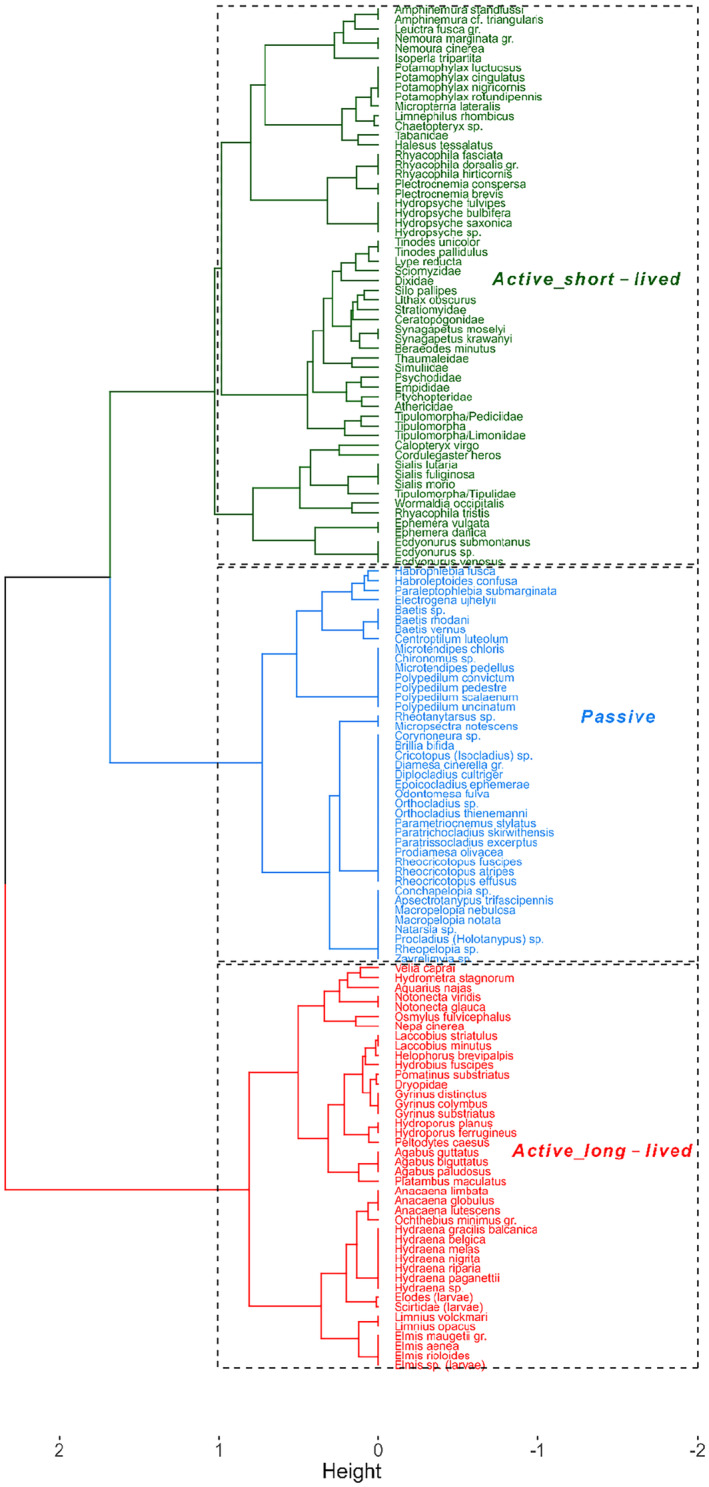
Hierarchical cluster analysis with Ward's method (using Gower dissimilarity metrics) showed three clusters: *Active long*‐*lived*, *Active short*‐*lived* and *Passive* dispersal groups.

Hierarchical cluster analysis showed three dispersal groups (hereafter DGs) representing different dispersal strategies of stream insects (Table 2 and Figures C1 and C2 in Appendix 3). ANOSIM confirmed the presence of three dispersal groups, showing relatively high dissimilarity among them (R = 0.66, *p* = .001). DGs were highly indicated by the trait categories of life cycle duration, adult life span, life‐long fecundity, dispersal strategy and propensity to drift based on multilevel pattern analysis (*r*_pb_ > 0.60; Table C2 in Appendix 3). The first DG (referred to as *Active long*‐*lived*) encompassed aerial active disperser taxa with long adult life span, small body size and low fecundity, which results in aerial active dispersal of individuals over short distances, but in multiple occasions. The second DG (referred to as *Active short*‐*lived*) also included aerial active dispersers, but with intermediate adult life span, medium to large body size, and intermediate fecundity resulting in aerial active dispersal of individuals over longer distances than the first DG, but more rarely done. The third DG (referred to as *Passive*) involved aerial passive dispersal with short adult life span, small body size and intermediate fecundity resulting in random dispersal of individuals in a limited number of occasions.

**TABLE 2:** Dispersal group characteristics, taxa belonging to and total number of taxa in each group.

Abbreviation	Description	Taxa	Number of taxa
Active long‐lived	*Long adult life span* *(* *>1 month* *)*; *long life cycle* *(* *>1 year* *)*; *low fecundity* *(* *<100 eggs* *)*; univotine; small female wings; small body size; aerial active disperser; propensity to drift: rare	Coleoptera, Heteroptera, Neuroptera	42
Active short‐lived	*Intermediate adult life span* *(* *>1 week to <1 month* *)*; *short life cycle* *(* *≤1 year* *)*; *intermediate fecundity* *(* *100–1000 eggs* *)*; semivoltine; medium female wings; medium to large body size; aquatic active disperser; propensity to drift: rare	Diptera (excl. Chironomidae), Ephemeroptera in part (Ephemeridae, Heptageniidae: *Ecdyonurus* spp.), Megaloptera, Odonata, Plecoptera, Trichoptera	58
Passive	*Short adult life span* *(* *<1 week* *)*, *short life cycle* *(* *<1 year* *)*; *intermediate* to high *fecundity* *(* *100–3000 eggs* *)*; multivoltine; small female wings; medium body size; *aerial passive disperser*; *propensity to drift*: *occasional*/frequent	Diptera: Chironomidae, Ephemeroptera in part (Baetidae, Leptophlebiidae, Heptageniidae: *Electrogena ujhelyii*)	41

*Note*: Highly indicative trait categories were highlighted with italic.

### 3.2 OFD patterns of stream insects

**FIGURE 2:**
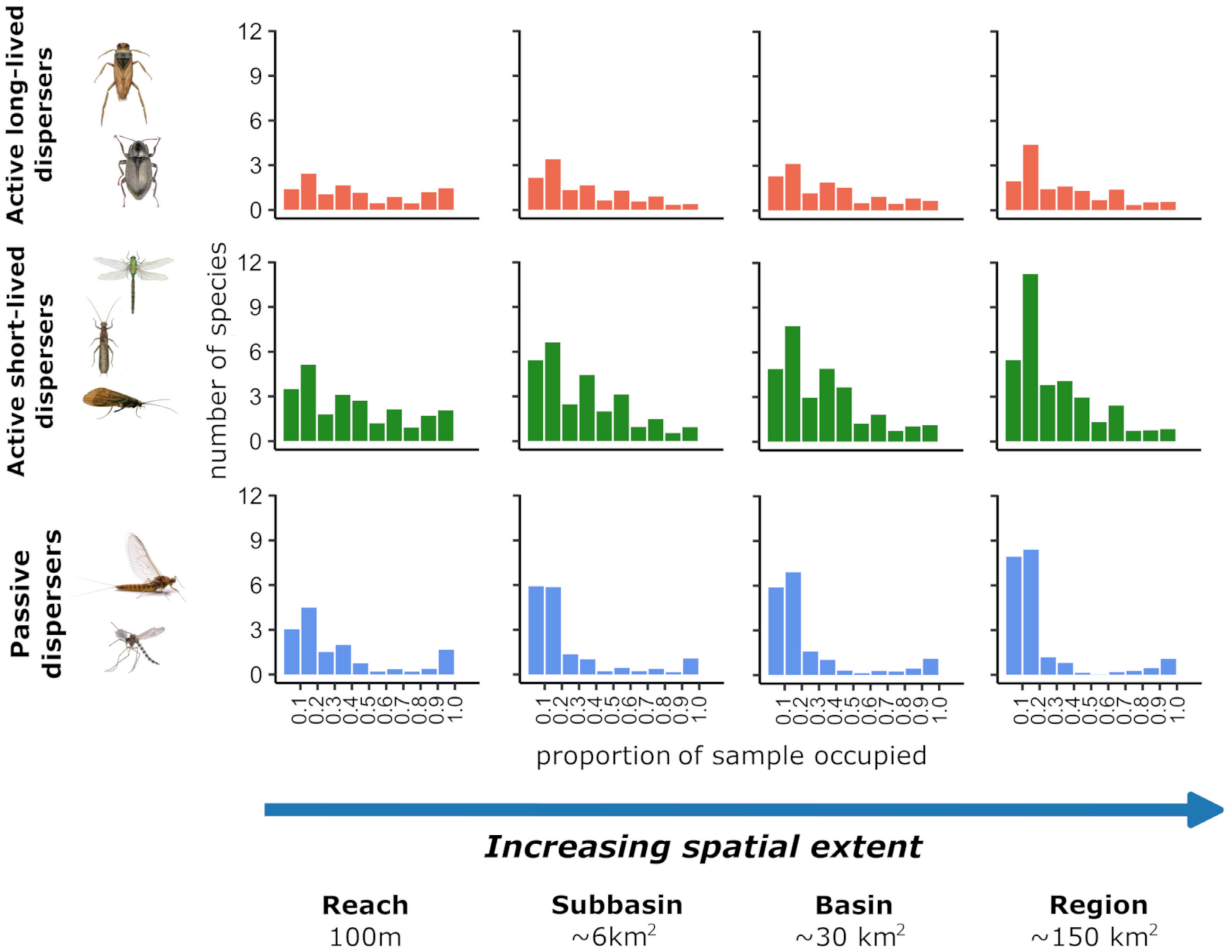
The average histograms of the occupancy frequency distribution (OFD) for stream insects were deconstructed based on three dispersal strategies and four spatial extents. The bar plots represent the mean values of numbers of taxa, calculated from 10,000 spatially representative samples (SRSs) for each dispersal strategy and spatial extent.

In total, 19,720 individuals belonging to 141 taxa were included in the analyses. Differences and similarities in the form of OFDs were observed among species groups with different dispersal strategies (Figure 2). Specifically, *Active long*‐*lived* DG exhibited varying occurrences, ranging from restricted to wide distribution classes. In the case of *Active short*‐*lived* DG, the species number in the left‐most classes (0–20% occurrence) was higher than in other classes, indicating a high number of rare species. However, OFD patterns also exhibited wide variability in the occurrence of higher distribution classes. Meanwhile, *Passive* DG displayed strongly right‐skewed OFD patterns with the dominance of rare species (0–20% class) at each spatial extent. Besides, as a general pattern for all dispersal groups, the number of rare species in the left‐most classes increased with the expanding spatial extent.

### 3.3 Effect of spatial extent on OFD patterns

**FIGURE 3:**
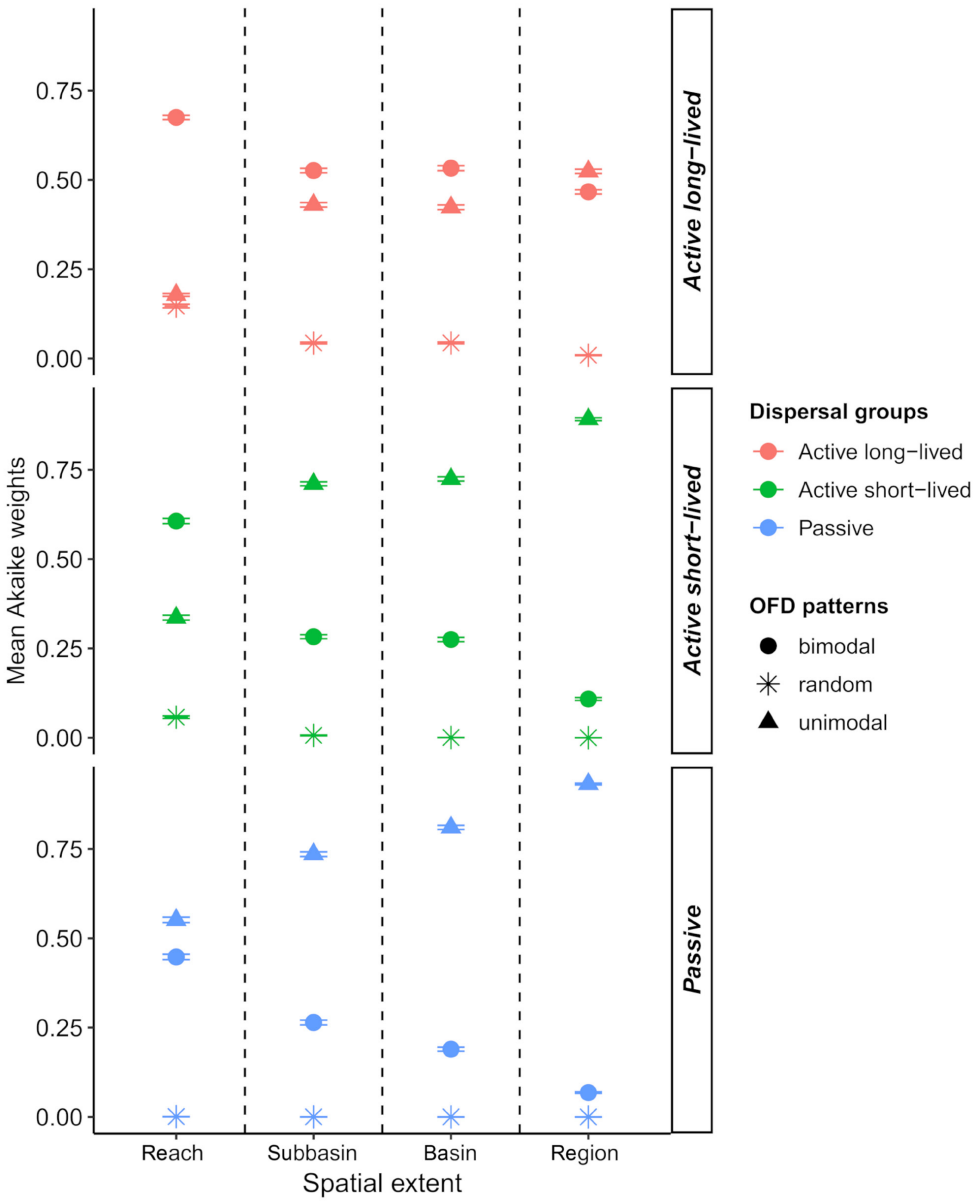
Spatial changes in the probability of unimodal, bimodal, and random OFD patterns. The mean Akaike weights of OFD patterns plotted against the increasing spatial extents separately for each dispersal group. Error bars represent the 95% confidence intervals. The names of the dispersal groups are defined in Table 2. The plot indicates that with increasing spatial extent, the OFD pattern changed from bimodal to unimodal for *Active long*‐*lived* and *Active short*‐*lived* DGs, but for *Passive* DG the probability of unimodal pattern is higher than that of the bimodal pattern at every spatial extent. The probability of the random pattern is negligibly low in all cases.

Based on Akaike weights, in most cases the probability of the unimodal pattern was higher than that of the bimodal pattern, whose high probability (*w* > 0.6) was only detected at the reach extent (Figure 3 and Table 3). On the contrary, we found high probability for unimodal pattern (*w* > 0.7) at subbasin, basin and regional extents for two dispersal groups (*Active short*‐*lived* and *Passive* DGs; Figure 3 and Table 3). The probability of the random pattern (*w* < 0.15) was negligibly low in all cases (Figure 3).

**TABLE 3:** The mean Akaike weights of OFD models (*unimodal*, *bimodal*, and *random* patterns), evidence ratios for OFD pattern pairs (BI/UNI, bimodal/unimodal; UNI/BI, unimodal/bimodal; RA/BI, random/bimodal; RA/UNI, random/unimodal), and the mean values of taxa number for different dispersal groups (*Active long*‐*lived*, *Active short*‐*lived*, *Passive* DGs) derived from samples with different spatial extents (*reach*, *sub* *basin*, *basin*, *region*). The names of the dispersal groups are defined in Table 2.

	Mean Akaike weight			Evidence ratio				Mean taxa num
	*Bimodal*	*Unimodal*	*Random*	BI/ UNI	UNI/BI	RA/BI	RA/UNI	
*Active long*‐*lived DG*								
Reach	0.67	0.18	0.15	3.82	0.26	0.22	0.84	12.07
Subbasin	0.52	0.43	0.04	1.21	0.83	0.08	0.10	12.81
Basin	0.53	0.43	0.05	1.22	0.82	0.09	0.11	13.45
Region	0.46	0.53	0.01	0.88	1.13	0.02	0.02	14.21
*Active short*‐*lived DG*								
Reach	0.62	0.33	0.05	1.85	0.54	0.08	0.16	24.23
Subbasin	0.28	0.71	0.01	0.40	2.51	0.02	0.01	28.12
Basin	0.27	0.73	7.76E‐04	0.37	2.73	2.90E‐03	1.06E‐03	30.35
Region	0.11	0.89	6.78E‐08	0.12	8.15	6.21E‐07	7.62E‐08	33.49
*Passive DG*								
Reach	0.44	0.56	9.40E‐04	0.79	1.26	2.13E‐03	1.69E‐03	14.78
Subbasin	0.26	0.74	2.54E‐04	0.36	2.81	9.66E‐04	3.44E‐04	16.83
Basin	0.19	0.81	7.91E‐05	0.23	4.32	4.20E‐04	9.74E‐05	17.76
Region	0.07	0.93	3.64E‐07	0.08	13.32	5.21E‐06	3.91E‐07	20.42

**FIGURE 4:**
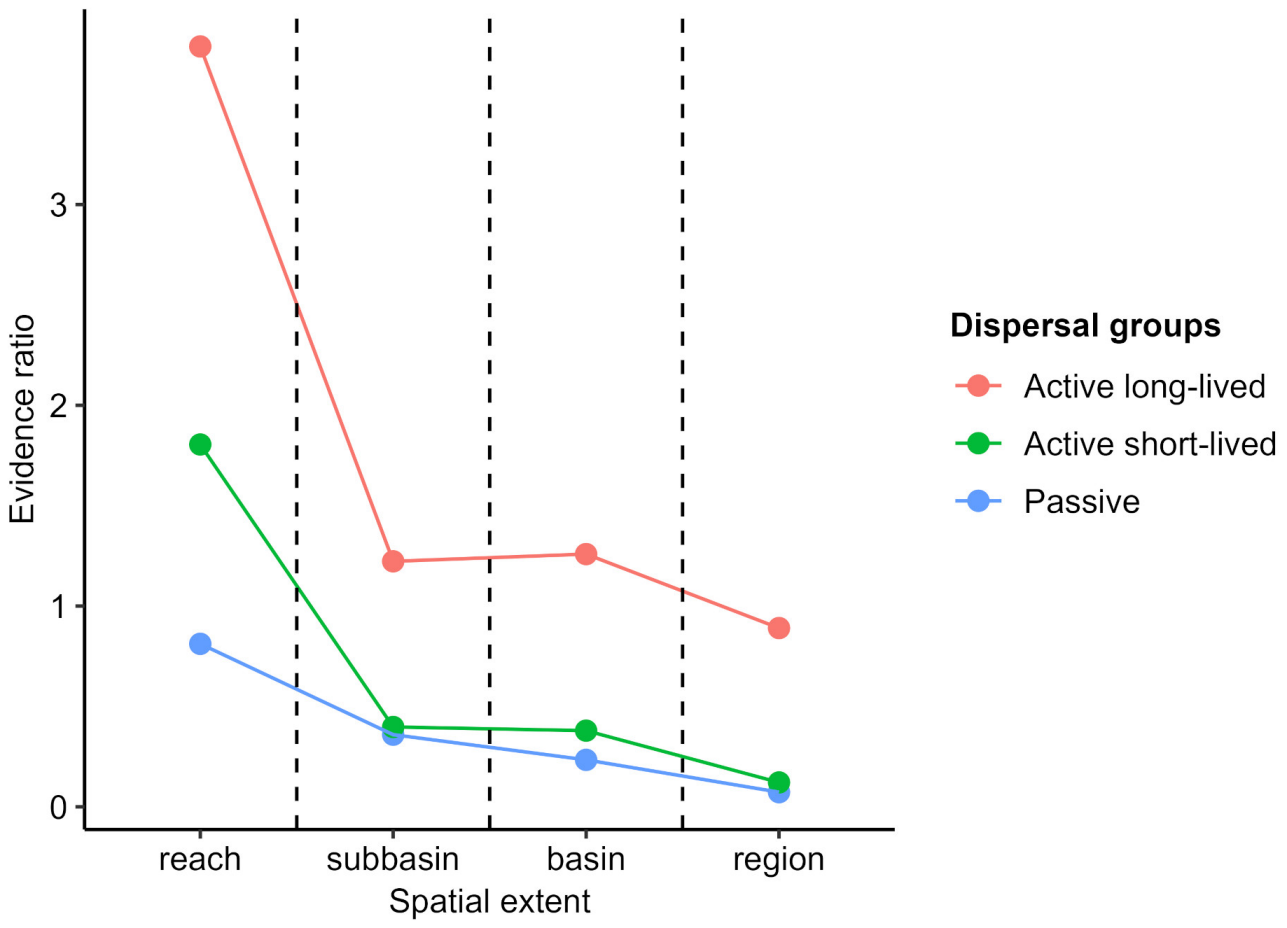
Evidence ratio (i.e. bimodal to unimodal, derived from mean Akaike weights for bimodal and unimodal OFD patterns) plotted against the increasing spatial extents, separately for each dispersal group. The names of the dispersal groups are defined in Table 2. Plot shows how the level of support for bimodal relative to unimodal patterns changes across spatial extents and dispersal groups. The level of support for bimodal patterns notably decreased relative to unimodal patterns with increasing spatial extent, and insect groups with different dispersal strategies differed in the strength of support for bimodal over unimodal OFD patterns across spatial extents.

The evidence ratio of Akaike weights indicated that the level of support for bimodal pattern notably decreased compared to unimodal pattern with increasing spatial extent (Figure 4). We observed a substantial change between reach and subbasin extents. For instance, in the case of *Active long*‐*lived* DG, the bimodal pattern was 3.7 times more likely to be the best model than the unimodal one at the reach extent, while this value was only 1.2 at the subbasin extent (Figure 4). In the case of *Active short*‐*lived* DG, the bimodal pattern was 1.8 times more likely than the unimodal one at the reach extent, whereas the support for the bimodal pattern was negligibly low (*ER* = 0.4) at the subbasin extent (Figure 4).

### 3.4 Effect of stream insect dispersal strategies

**FIGURE D1:**
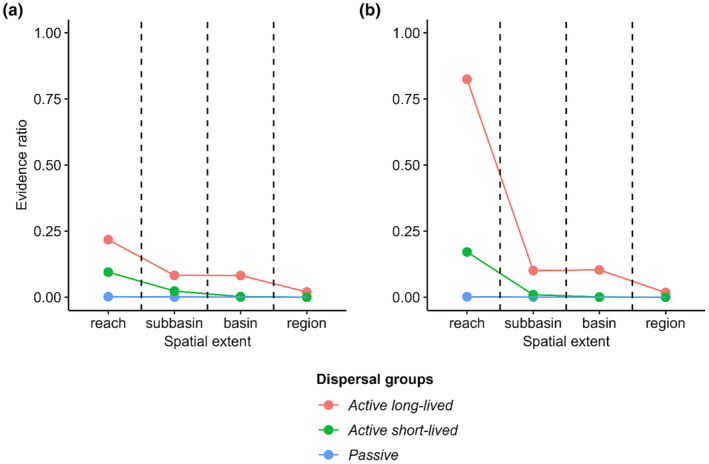
Evidence ratio derived from mean Akaike weights of (a) random‐bimodal and (b) random‐unimodal OFD pattern pairs plotted against the increasing spatial extents separately for each dispersal group. The names of the dispersal groups are defined in Table 2. Plot shows how the level of support for random pattern over (a) bimodal or (b) unimodal one changes across spatial extents and dispersal groups.

Based on Akaike weights and evidence ratios, insect groups with different dispersal strategies differed in the strength of support for OFD patterns at all spatial extents (Figures 3 and 4 and Table 3). At the reach extent, the bimodal pattern was more likely to be favoured over the unimodal one for *Active long*‐*lived* DG compared to *Active short*‐*lived* DG; while for *Passive* DG the support for both OFD patterns as the best model was nearly similar (Figures 3 and 4 and Table 3). At this spatial extent, for *Active long*‐*lived* DG, the level of support for the random pattern was nearly similar to the uniform one but substantially weaker than the support for the bimodal pattern (Figures 3 and D1 in Appendix 4 and Table 3).

At the subbasin and higher extents, the *Active long*‐*lived* DG differed from the *Active short*‐*lived* and *Passive* DGs in terms of the support for OFD patterns. Evidence ratios provided higher support in the favour of the bimodal pattern over the unimodal one for *Active long*‐*lived* DG compared to the other two DGs (Figure 4 and Table 3). However, for *Active long*‐*lived* DG, the support for both bimodal and unimodal patterns as the best model was nearly similar (Figures 3 and 4). In contrast, the *Active short*‐*lived* and *Passive* DGs exhibited similarly weak support for the bimodal pattern over the unimodal one, which was much more likely to be the best model (Figure 4 and Table 3).

The evidence ratios of the different dispersal groups, expressing the strength of support for one OFD pattern versus others, changed with increasing spatial extent (Figure 3). For *Passive* DG, the support for the unimodal pattern was higher than the bimodal one and sharply increased across all spatial extents (Figure 4 and Table 3). *Active short*‐*lived* DG exhibited a conversion point between the reach and subbasin extents, where the previously more likely preferred bimodal pattern became weakly supported, while the previously unlikely unimodal pattern became strongly supported over their competitors (Figure 4 and Table 3). The *Active long*‐*lived* DG also exhibited a substantial decline in the strength of support for the bimodal pattern over the unimodal one from reach to subbasin extent. The bimodal pattern obtained lower support compared to the unimodal one only at the regional extent (Figures 3 and 4).

### 3.5 Effect of sampling parameters

**FIGURE 5:**
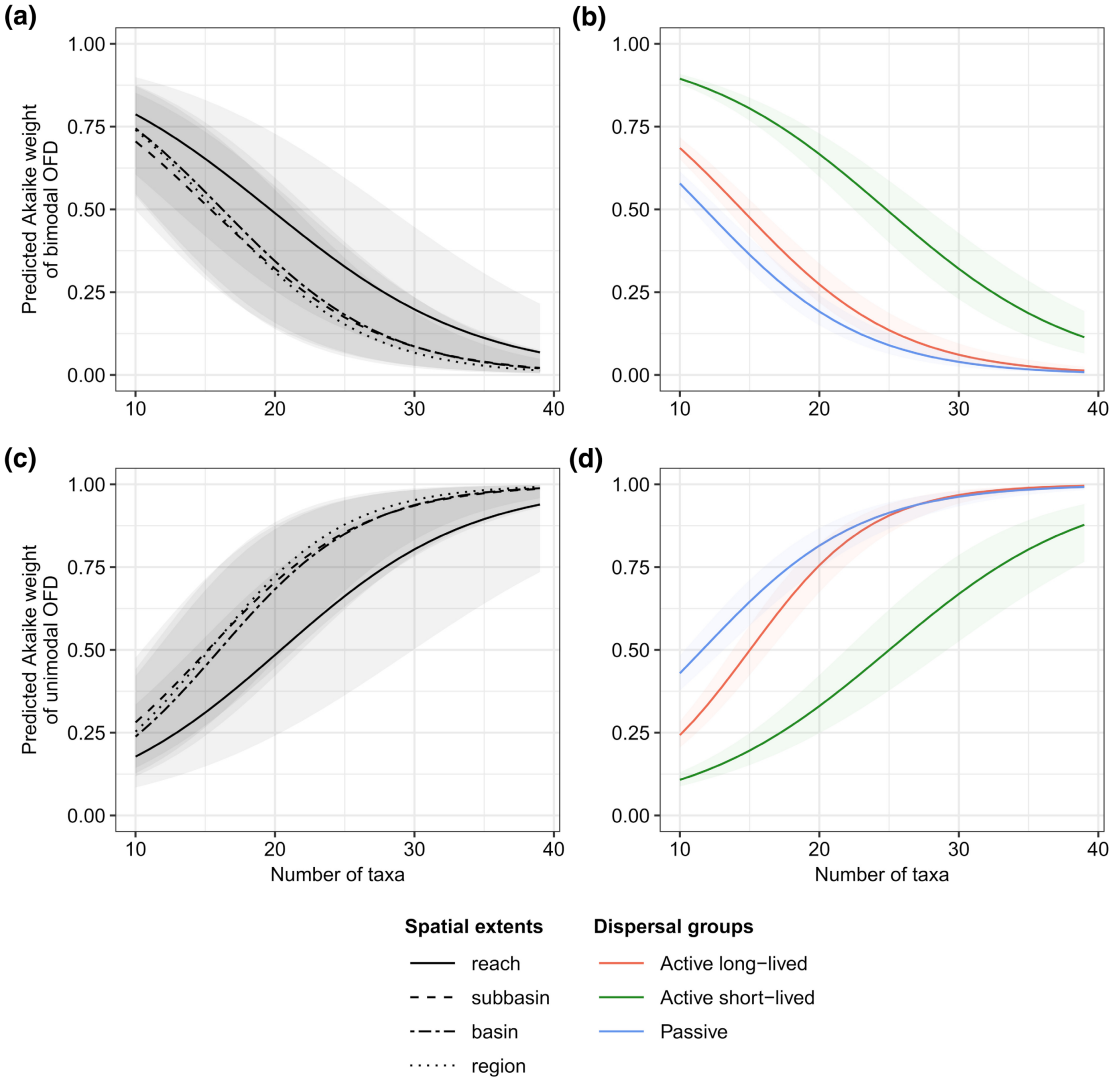
The response curves of Akaike weights of bimodal (a, b) and unimodal (c, d) OFD patterns as a function of taxa number based on Generalised Linear Mixed Models. We examined the joint effect of taxa number and spatial extent (a, c), as well as the interaction of taxa number and dispersal group (b, d) on the Akaike weights of OFD patterns in separate regression models. With increasing taxa richness, the probability of the bimodal patterns significantly decreases, and the probability of unimodal pattern significantly increases within each spatial extent and dispersal group.

**FIGURE E1:**
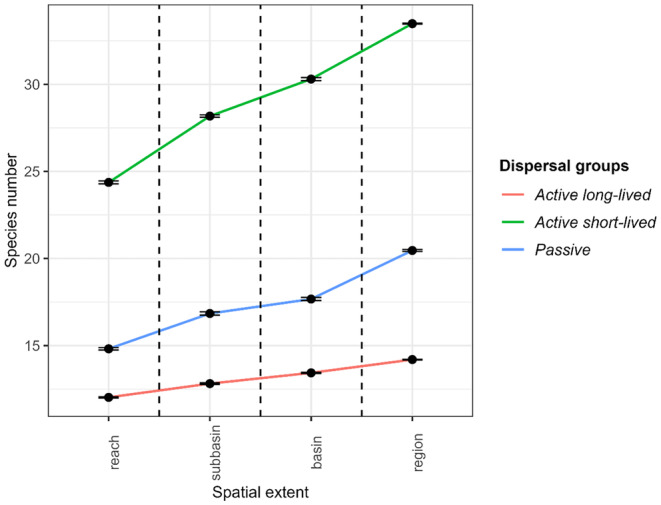
Changes in the number of taxa along increasing spatial scale. The mean number of taxa plotted against the increasing spatial scale separately for each dispersal group. Error bars represent the 95% confidence intervals. The names of the dispersal groups are defined in Table 2.

We found a higher number of taxa for *Active short*‐*lived* DG than for other two dispersal groups at each spatial extent; while the lowest number of taxa characterised the *Active long*‐*lived* DG (Table 3 and Figure E1 in Appendix 5). The GLMMs indicated significant influence of the main and the interaction effects of the taxa number and spatial extent, as well as the taxa number and dispersal group on the probability of both OFD patterns (Table E1 in Appendix 5). The predicted slope of GLMM curves indicated a positive relationship (predicted slopes changed between 0.141 and 0.227) between taxa number and the probability of unimodal pattern, as well as a negative association (predicted slopes between −0.135 and −0.184) with the probability of bimodal pattern within each spatial extent and dispersal group (Figure 5 and Table E2 in Appendix 5). The strength of these relationships was significantly weaker at the reach extent than at larger spatial extents (Figure 5 and Table E2 in Appendix 5). The *Active short*‐*lived* DG showed a gentler predicted slope of GLMM curves compared to the other two dispersal groups, implying the weakest relationship between the probability of bimodal and unimodal patterns and taxa number (Figure 5 and Table E2 in Appendix 5). The intercepts of GLMM curves indicated that for a given taxa number the probability of bimodal patterns tended to be highest at subbasin extent, while the probability of unimodal pattern was lowest at subbasin extent compared to other spatial extents (Figure 5 and Table E2 in Appendix 5). In the case of *Active short*‐*lived* DG, for a given taxa number, the probability of bimodal patterns was significantly higher than for *Active long*‐*lived* and *Passive* DGs, whose probability values did not differ from each other. While for a given taxa number the probability of unimodal pattern was highest for *Passive* DG compared to other two DGs, whose probability values did not differ from each other (Figure 5 and Table E2 in Appendix 5).

**FIGURE E2:**
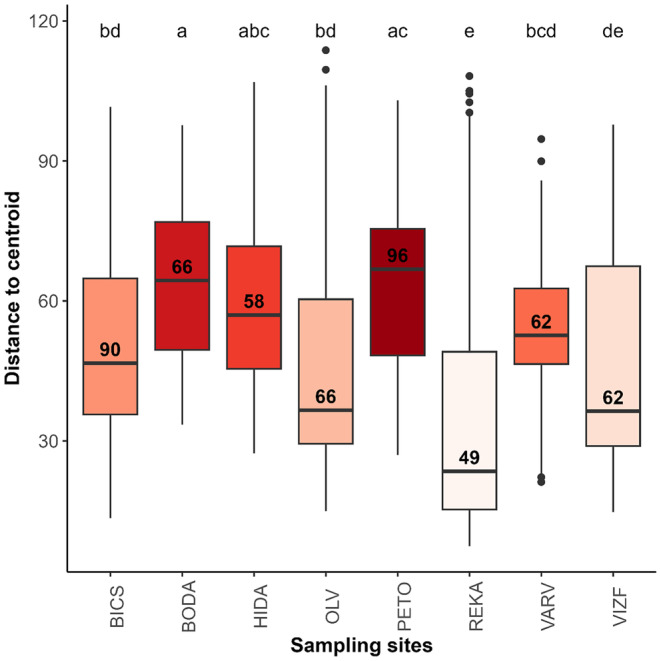
The results of the post‐hoc Tukey HSD test of PERMDISP analysis on the percentage coverage of substrate types and the sampling sites. The box‐plots indicate the microhabitat heterogeneity within reaches. Letters above the box‐plots indicate significantly different groups regarding microhabitat heterogeneity between sampling sites, provided that there is no letter simultaneously present in two given groups. The numbers in box‐plots indicate the annual cumulative taxa number for each reaches. The intensity of the red colour in the boxplots indicates the level of microhabitat heterogeneity.

Based on PERMDISP analysis, we found significant differences in microhabitat heterogeneity among the sampling reaches (df = 7, *F* = 14.596, *p* < .001). Although, the post‐hoc Tukey HSD tests indicated that there were differences in microhabitat heterogeneity among reaches, but none of them completely distinguished itself from all the others (Figure E2 in Appendix 5). Finally, based on Spearman's rank correlation tests we did not find significant relationship between the microhabitat heterogeneity of reaches and their annual cumulative taxa number (rho = 0.482, *p* = .23).

## 4 DISCUSSION

We could confirm our first prediction in which the OFD pattern changed from bimodal to unimodal with increasing spatial extent. There was a general tendency across all dispersal groups for the level of support for unimodal pattern to increase over other OFD patterns with increasing spatial extent. This result reflected the underlying changes in the relative role of the metacommunity processes (i.e. dispersal and niche processes) structuring stream insect assemblages if the spatial extent of sampling was expanded from the reach to the regional extent. In coincidence with our second expectation, the use of combinations of dispersal traits was suitable for revealing differences in OFD patterns among insect groups with different dispersal strategies, particularly because contrasting dispersal groups differed in the strength of support for OFD patterns at all spatial extents. At the reach extent, the bimodal pattern tend to be more favoured for active dispersal insect groups, while the unimodal pattern was more supported for the passive dispersal group. Conversely, at the subbasin and larger spatial extents, there was strong evidence supporting the unimodal pattern as the best model for both *Active short*‐*lived* and *Passive* DGs. For *Active long*‐*lived* DGs, the support for both bimodal and unimodal patterns was nearly equal. Furthermore, the strength of support for OFD patterns varied across dispersal groups differently as the spatial extent increased. Our findings suggest that the stream insect dispersal strategy influences the relative role of dispersal and niche processes particularly as spatial extent increases from stream reaches to the extent of adjacent valleys.

We could not rank the three dispersal groups regarding the strength of their dispersal and colonisation. Consistent with other studies, we found that some dispersal‐related traits showed trade‐offs with each other (e.g. adult life span and fecundity; Schmera et al., 2022; Verberk et al., 2008, 2010; Wilkes et al., 2020). This implies that for successful colonisation, if some traits of species are weak (e.g. small body size) regarding dispersal ability, then species are strong in other traits that support dispersal or colonisation (e.g. high fecundity). For instance, it is well known that active dispersers have higher dispersal effectiveness via direct movement than passive ones (e.g. Sarremejane et al., 2020). However, in our study, *Passive* DG also showed intermediate fecundity and short generation time (multivoltinism), which promoted their colonisation success (see also Verberk et al., 2008). For *Active* DGs, we detected trade‐offs among adult life span and body size or female wing length (Table C2 in Appendix 3). Specifically, the *Active short*‐*lived* DG had lower dispersal and colonisation success due to a short adult life span (Sarremejane et al., 2020). However, a single long‐distance event by a large‐sized and gravid female could promote their colonisation of new habitats (Tonkin, Heino & Altermatt, 2018b). Consequently, we assume that the three dispersal groups exhibit similar dispersal success but represent different dispersal strategies.

In agreement with previous studies (Collins & Glenn, 1991, 1997; Heatherly et al., 2007; Suhonen & Jokimäki, 2019), our results supported the spatial scale dependence of the OFDs of freshwater insects as predicted by the distance scaling principle. For *Active short*‐*lived* DG, the OFD pattern changed from bimodal to unimodal with increasing spatial extent (Figure 4). For *Active long*‐*lived* DG, the support for the bimodal pattern over the unimodal one notably declined from reach to subbasin extent. The support for both OFD patterns was nearly equal at larger spatial extents. McGeoch and Gaston (2002) argued that this shift suggested underlying changes in mechanisms structuring assemblages along an increasing spatial extent.

In our study at the reach extent, the high support for the bimodal pattern indicated that all habitat patches could potentially be available to all taxa (Hui, 2012). Therefore, we suggest that dispersal limitation did not determine the organisation of communities, but rather metapopulation dynamics (Hanski, 1982) or the mass effect (Leibold et al., 2004). In temperate streams, habitat patches are highly exposed to frequent hydrological disturbances, which greatly reduce or eliminate individuals from their habitats (Death, 2010; Dodds et al., 2004; Reice et al., 1990). At the same time, the recolonisation of organisms from adjacent patches could also be a continuous process (Mehranvar & Jackson, 2001). If we assumed that species perceived habitat patches to be of similar quality, the occupancy dynamics of insect species could be balanced by the source‐sink dispersal as suggested by the metapopulation dynamic model (Hanski, 1982). Alternatively, if species perceived habitat patches to be of different quality, the high dispersal rate of species at reach extent enabled them to occur in suboptimal habitat patches and could become common in a stream reach (mass effect; Leibold et al., 2004).

At regional extent in Mecsek Mountains, our results indicated strong evidence for the unimodal pattern that agreed with many other studies in freshwater ecology (e.g. Heino, 2008, 2015; Malmqvist et al., 1999; Renner et al., 2020; Suhonen, 2021). Previous studies have confirmed that the effectiveness of species dispersal decreased, and the environmental heterogeneity increased with increasing spatial extent (Collins & Glenn, 1997; McGeoch & Gaston, 2002). We must also consider the influence of growing taxa richness along increasing spatial extent on the changes of OFD patterns, because it showed a significant positive relationship with the probability of unimodal pattern (Figure 5 and Tables E1 and E2 in Appendix 5). However, the strength of this relationship was weaker (Table E2 in Appendix 5), and for the range of the relevant taxa richness, the probability of the unimodal pattern was lower at reach extent than at larger spatial extents (Figure 5). This might contribute to the more enhanced probability of the unimodal OFD pattern mainly in larger spatial extents (McGeoch & Gaston, 2002). These and other factors (e.g. the sampling area is located in a biogeographic transition zone) could jointly limited the occupancy of species, resulting in many sites with high ratio of rare species. Thus, besides increasing taxa richness, we assume that niche processes jointly with dispersal limitation could be important mechanisms structuring the stream insect assemblages at regional (approx. 150 km^2^) and larger spatial extent.

Within a given spatial extent, insect groups with contrasting dispersal strategies showed differences in the support for OFD patterns (Figure 4). When discussing the effect of dispersal groups on the OFD patterns, it is important to assess that taxa richness notably varied among them at all spatial extents (Figure E1 in Appendix 5), and taxa richness is significantly positively correlated with the probability of unimodal pattern, and significantly negatively correlated with the probability of bimodal pattern (Tables E1 and E2 in Appendix 5). However, our results indicated differences in the strength of the relationship between taxa richness and the probability of OFD patterns, as well as differences in the probability of unimodal and bimodal OFD patterns for a given taxa richness among dispersal groups (Tables E2 and E3 in Appendix 5). Specifically, for a given taxa richness *Active short*‐*lived* DG had higher probability of bimodal pattern than other DGs, while *Passive* DG had higher probability of unimodal pattern than other DG (Figure 5 and Tables E2 and E3 in Appendix 5). Consequently, we concluded that our results supported the prediction of the organismal scaling principle (Collins & Glenn, 1997; Suhonen et al., 2022).

At the reach extent, strong support for bimodal pattern was detected for *Active* DGs, while the unimodal and bimodal patterns were nearly similarly supported for *Passive* DG (Figure 4). Based on our results, assuming equal taxa richness for all dispersal groups, mirroring the group with the lowest number of taxa at the reach extent (i.e. 12.07 taxa in *Active*‐*long lived* DG; Table 3), our findings would remain unchanged, except for a stronger support for bimodal patterns in the case of *Active short*‐*lived* DG (Table E3 in Appendix 5). Considering that *Passive* DG includes medium‐sized drifting species, we assumed that all habitat patches could potentially be available to them as they are to *Active* DGs. Consequently, in the case of *Passive* DG the unimodal pattern might rise from the unique environmental preference of taxa.

At the subbasin and larger extents, there was stronger support for the bimodal pattern over the unimodal pattern in *Active long*‐*lived* DG compared to the other two dispersal groups, where the unimodal pattern was more likely to be favoured (Figure 4). Our results may arise from the lowest taxa richness of the *Active long*‐*lived* DG (Table 3) and the negative correlation between taxa richness and the probability of bimodal OFD (Figure 5 and Table E2 in Appendix 5). If we presume equal taxa richness for all DGs, mirroring the group with the lowest number of taxa at larger spatial extents (i.e. 12.81–14.21 taxa in *Active*‐*long lived* DG; Table 3), the shift from bimodal to unimodal would occur at a smaller spatial extent, not for the *Active short*‐*lived* DG but for the *Active long*‐*lived* DG (Table E3 in Appendix 5). Therefore, we assumed that the dispersal capacity of the taxa in *Active short*‐*lived* DG could be high enough to maintain a significant number of core species in the case of larger spatial extents. While for *Passive* DG and presumably, for *Active long*‐*lived* DG, the effectiveness of dispersal might reduce to such an extent across adjacent valleys and larger spatial extents leading to the dominance of rare species and thus likely enhancing the potential role of niche processes (Brown et al., 2011; Brown, 1984).

Different dispersal groups differed regarding the changes in the strength of support for the one OFD pattern versus others with increasing spatial extent (Figure 4). For *Passive* DG, the support for the bimodal pattern decreased to a similar extent along increasing spatial extent as predicted by the GLMM models (Table E3 in Appendix 5) in relation to the observed taxa richness intervals (14.78–20.42 taxa; Table 3). While, in the case of the two *Active* DGs, the support for the bimodal pattern decreased to a higher intensity between reach and subbasin extents than across larger spatial extents (Figure 4). In relation to the observed taxa richness intervals (Table 3), we found that this reduction between reach and subbasin extent was nearly 10 (*Active long*‐*lived* DG) and three (*Active short*‐*lived* DG) times stronger than what the GLMM models predicted (Table E3 in Appendix 5). Consequently, based on these results we suggest that the dispersal strategy of organisms could influence the relative role of dispersal and niche processes, particularly as spatial extent increases from stream reaches to the extent of adjacent valleys (Tonkin, Heino & Altermatt, 2018b).

Finally, we also need to consider the effect of environmental heterogeneity when interpreting our results. In our study, there was a significant difference in microhabitat heterogeneity among reaches; however, none of them distinctly separated itself from all the others, and there was no significant relationship between microhabitat heterogeneity and cumulative taxa richness of reaches. At the reach extent, when environmentally more homogeneous reaches (e.g. REKA) were selected into a spatially representative sample (SRS) during the re‐sampling procedure, it might give a higher probability to the bimodal pattern. However, due to the 10,000 repetitions, reaches with high environmental heterogeneity were also included in the reach extent sample, increasing the probability of the unimodal pattern. Thus, thanks to the 10,000 repetitions, the effect of more homogeneous habitats was balanced with the effect of more heterogeneous ones. At the subbasin and larger spatial extents, during the re‐sampling procedure, sample units were evenly selected from 3, 4, and 8 reaches. This way, a spatially representative sample (SRS) was composed of sample units from reaches with various levels of heterogeneity, providing a general result for the support of different OFD patterns, which encompassed reaches with different levels of environmental heterogeneity. Consequently, we suppose that our sampling design could mask the effect of environmental heterogeneity on the probability of OFD patterns.

In agreement with previous studies, we found negligible support for random pattern in almost all cases, which was exceeded only by the *Active long*‐*lived* DG at the reach extent (Jenkins, 2011; McGeoch & Gaston, 2002). Jenkins (2011) argued that random patterns might be observed in assemblages under transient conditions, such as at an early stage in the colonisation history of habitats. In small streams, this transient stage could occur because of the effects of frequent floods or droughts (Death, 2010). Floods might wash out small insects such as the members of *Active long*‐*lived* DG; however, being fast colonisers could reach these vacant habitats from adjacent streams in a short time. Our results suggest that the early recolonisation processes might contribute to shaping the OFD pattern at a small spatial extent.

## 5 CONCLUSIONS

We described the role of spatial extent and dispersal strategy in shaping the OFD patterns of insect assemblages inhabiting near‐pristine temperate streams. The novelty of our study is that we could define spatial extents and dispersal strategies within which unique metacommunity processes are likely underlie the organisation of assemblages. Nevertheless, we agree with Collins and Glenn (1997), who suggested that the general validity or utility of a given model is limited.

In concordance with Verberk et al. (2008, 2010), we also concluded that the use of a combination of dispersal traits is suitable for finding more general ecological patterns, since it reduces many complex, diverse assemblages to a few meaningful relationships. Our results are consistent with the opinion of Tonkin, Altermatt et al. (2018a) that considering dispersal strategies gives a more complete view of the relative role of dispersal and niche processes in shaping metacommunities along a riverine network.

The general pattern in our study is the high number of satellite species in every spatial extent, which corresponds to many other studies on different ecosystems, ecoregions and taxonomical groups (see in McGeoch & Gaston, 2002). It follows that the high amount of rarity (i.e. taxa with rare regional occurrence) is a general and robust characteristic of near‐natural ecosystems. The high number of satellite species can contribute to functional redundancy, which is the principal mechanism by which communities recover after disturbances and, consequently, increase the resilience of ecosystems (Angeler & Allen, 2016; Brown, 1984; Van Looy et al., 2019). In the light of the global climate change, the large‐scale diversity loss, and the invasion of alien species, it is crucial to better understand how strong disturbances can reduce or demolish the dominance of rarity in freshwater ecosystems.

## AUTHOR CONTRIBUTIONS

**I. Szivák:** Conceptualization (equal); data curation (lead); formal analysis (equal); investigation (lead); methodology (equal); visualization (lead); writing – original draft (lead). **Z. Csabai:** Resources (equal); supervision (lead); writing – review and editing (equal). **D. Schmera:** Formal analysis (equal); funding acquisition (lead); methodology (supporting); resources (equal); writing – review and editing (equal). **A. Móra:** Conceptualization (equal); methodology (equal); writing – original draft (supporting); writing – review and editing (equal).

## FUNDING INFORMATION

The research was funded by the National Multidisciplinary Laboratory for Climate Change (RRF‐2.3.1‐21‐2022‐00014) and by the Sustainable Development and Technologies National Programme of the Hungarian Academy of Sciences (FFT NP FTA, NP2022‐II3/2022).

## CONFLICT OF INTEREST STATEMENT

The authors declare no conflicts of interest.

## Data Availability

All data and scripts supporting the results and analyses can be accessed on Zenodo at https://doi.org/10.5281/ZENODO.8125637.

## APPENDIX 1

### 1.1 Detailed information for sampling sites

**TABLE A1:** The abbreviations, names and geo‐coordinates of sampling sites.

Abbrev.	Name (municipality)	N	E
BICS	Bicsérdi stream (Bakonya)	46.08708	18.09136
BODA	Bodai stream (Boda)	46.08669	18.05386
HIDA	Hidasi stream (Komló)	46.19419	18.31772
OLV	Ól valley (Szászvár)	46.26353	18.36722
PETO	Petőczi stream (Bakonya)	46.12127	18.05338
REKA	Réka valley (Mecseknádasd)	46.22141	18.43472
VARV	Vár valley stream (Magyaregregy)	46.22761	18.32205
VIZF	Vízfő stream (Orfű)	46.13907	18.16164

## APPENDIX 2

### 2.1

The keys used for the identification of the freshwater insect groups.

## APPENDIX 3

### 3.1 Dispersal‐related morphological, behavioural and life‐history traits based on which taxa were classified to contrasting dispersal groups

**TABLE C1:** Selected traits and the rationale for their use as indicators of the dispersal potential of species.

Trait	Categories	Rationale based on Sarremejane et al. (2020)
*Morphology*		
Body size	Small (<0.5 cm)	*‘Maximum body size influences organisms' dispersal*, *especially for active dispersers*, *with larger animals more capable of active dispersal over longer distances’*
	Medium (0.5–2 cm)	
	Large (2–8 cm)	
Female wing length	None	*‘Females connect and sustain populations through oviposition*, *thus representing adult insects' colonisation capacity*. *Females with larger wings are likely to oviposit farther from their source population’*
	Small (<10 mm)	
	Medium (10–30 mm)	
	Large (30–60 mm)	
*Behaviour*		
Dispersal strategy	Aquatic passive	Represent a taxon's predominant dispersal mode: active disperser capable of dispersal over longer distances compared by passive disperser
	Aquatic active	
	Aerial passive	
	Aerial active	
Propensity to drift	Rare	*‘indicates the frequency of flow*‐*mediated passive downstream dispersal events’*
	Occasional	
	Frequent	
*Life*‐*history traits*		
Life span		
Life cycle duration	<1 year	*‘Reflect the adult* (i.e. *reproductive*) *and total life duration*, *with longer*‐*lived animals typically having more dispersal opportunities’*
	>1 year	
Adult life span	<1 week	
	1 week–1 month	
	1 month–1 year	
	>1 year	
Potential propagule production		
Reproductive cycles per year	<1	*‘Assess dispersal capacity based on potential propagule production*, *with multiple reproductive cycles and abundant eggs typically increasing the number of dispersal events’*
	1	
	>1	
Life‐long fecundity (no. of eggs per female)	<100	
	100–1000	
	1000–3000	
	>3000	

### 3.2 The results of multilevel pattern analysis which shows highly indicative trait categories for each dispersal groups

**TABLE C2:** Indicator value (as point‐biserial coefficients; *r*_pb_) and corresponding *p*‐value for each trait category in each dispersal group (*Active long*‐*lived*, *Active short*‐*lived*, *Passive*) and their combinations.

Trait	Trait categories	*Active long*‐*lived*	*Active short*‐*lived*	*Passive*	*r*_pb_	*p*‐value
Body size	Small (<0.5 cm)	1			0.53	0.001
	Medium (0.5–2 cm)		1	1	0.39	0.001
	Large (2–8 cm)		1		0.47	0.001
Female wing length	Small (<10 mm)	1		1	0.53	0.001
	Medium (10–30 mm)		1		0.58	0.001
	Large (30–60 mm)		1		0.15	0.338
Dispersal strategy	Aquatic passive			1	0.40	0.001
	Aquatic active	1	1		0.20	0.043
	Aerial passive			1	0.66	0.001
	Aerial active	1			0.52	0.001
Propensity to drift	Rare	1	1		0.48	0.001
	Occasional			1	0.61	0.001
	Frequent			1	0.56	0.001
Life cycle duration	<1 year		1	1	0.65	0.001
	>1 year	1			0.75	0.001
Adult life span	<1 week			1	0.84	0.001
	1 week–1 month		1		0.72	0.001
	1 month–1 year	1			0.75	0.001
	>1 year	1			0.70	0.001
Reproductive cycles per year	<1		1		0.51	0.001
	1	1			0.40	0.001
	>1			1	0.50	0.001
Life‐long fecundity (no. of eggs per female)	<100	1			0.77	0.001
	100–1000		1	1	0.59	0.001
	1000–3000			1	0.34	0.001
	>3000		1		0.20	0.039

*Note*: ‘1’ indicates the dominant trait category in each dispersal group. The *r*_pb_ value is proportional to the occurrence of the trait category in the dispersal group. The names of the dispersal groups are defined in Table 2.

## APPENDIX 5

### 5.1 Effect of taxa richness on the Akaike weights of bimodal and unimodal OFD pattern

**TABLE E1:** ANOVA table output of GLMMs examining the individual and joint effects of predictors on Akaike weights.

	Unimodal OFD			Bimodal OFD		
	LR Chisq	df	*p*‐value	LR Chisq	df	*p*‐value
*Taxa number* × *spatial extent*						
Taxa number	76.3	1	<0.001	463.0	1	<0.001
Spatial extent	9393.5	3	<0.001	4873.2	3	<0.001
Taxa number *×* spatial extent	1408.6	3	<0.001	1032.0	3	<0.001
*Taxa number* × *dispersal group*						
Taxa number	231.0	1	<0.001	312.9	1	<0.001
Dispersal group	33391.5	2	<0.001	27479.7	2	<0.001
Taxa number *×* dispersal group	585.9	2	<0.001	244.6	2	<0.001

*Note*: We examined the effects of taxa number and spatial extent, as well as the influence of taxa number and dispersal group on the Akaike weights of unimodal (Unimodal OFD) and bimodal (Bimodal OFD) patterns in separate models.

**TABLE E2:** The summary table of coefficients of Analysis of Covariance using Generalised Linear Mixed Models (GLMM) conducted on the Akaike weights of OFD patterns for all spatially representative samples as response variables, taxa number for all spatially representative samples as continuous explanatory variables, and spatial extent, dispersal group as categorical explanatory variables. We involved individual assemblages within spatial extents and dispersal groups (i.e. assemblages defined by dispersal groups, and assemblages in each spatial extent, respectively) as random factors. The structure of summary table followed the output table of ‘summary()’ command in R program.

	Estimate	Std. error	*z* value	*p*‐value	Cum. est.
*Model*: *Akaike weights ~ taxa number×spatial extents*					
Unimodal OFD					
Intercept	−3.003	0.386	−7.770	<0.001	−3.003
Taxa number	0.147	0.020	7.230	<0.001	0.147
Subbasin extent	0.257	0.028	9.240	<0.001	−2.746
Basin extent	−0.096	0.028	−3.400	<0.001	−3.099
Regional extent	−0.120	0.032	−3.800	<0.001	−3.123
Taxa number: subbasin	0.034	0.001	23.020	<0.001	0.181
Taxa number: basin	0.046	0.001	31.480	<0.001	0.193
Taxa number: region	0.057	0.002	36.700	<0.001	0.204
Bimodal OFD					
Intercept	2.661	0.373	7.130	<0.001	2.661
Taxa number	−0.135	0.008	−17.160	<0.001	−0.135
Subbasin extent	−0.167	0.028	−5.880	<0.001	2.495
Basin extent	0.125	0.028	4.410	<0.001	2.787
Regional extent	0.225	0.031	7.260	<0.001	2.886
Taxa number: subbasin	−0.027	0.001	−18.030	<0.001	−0.162
Taxa number: basin	−0.036	0.001	−24.580	<0.001	−0.172
Taxa number: region	−0.049	0.002	−31.780	<0.001	−0.184
*Model*: *Akaike weights ~ taxa number×dispersal groups*					
Unimodal OFD					
Intercept	−3.526	0.086	−41.010	<0.001	−3.526
Taxa number	0.141	0.011	13.030	<0.001	0.141
*Active long*‐*lived* DG	0.120	0.065	1.830	0.067	−3.407
*Passive DG*	1.475	0.049	29.920	<0.001	−2.052
Taxa number:*Active long*‐*lived DG*	0.086	0.004	21.630	<0.001	0.227
Taxa number:*Passive* DG	0.036	0.002	17.810	<0.001	0.177
Bimodal OFD					
Intercept	3.586	0.087	41.220	<0.001	3.586
Taxa number	−0.145	0.009	−15.710	<0.001	−0.145
*Active long*‐*lived* DG	−1.050	0.066	−15.850	<0.001	2.536
*Passive* DG	−1.513	0.050	−30.570	<0.001	2.072
Taxa number:*Active long*‐*lived* DG	−0.031	0.004	−7.690	<0.001	−0.176
Taxa number:*Passive* DG	−0.031	0.002	−15.440	<0.001	−0.176

*Note*: The table presented the predicted values (*Estimate*), the standard errors (*Std*. *Error*), and the *z*‐test results (*z value*, *p*‐*value*) of the y‐axis intercepts of GLMM curves at the reference (*intercept*) and other levels of categorial explanatory variables (*sub* *b* *asin extent*, *basin extent*, *regional extent*, *Active long*‐*lived* DG, *Passive* DG), and the slope of GLMM curves at the reference (*taxa number*) and other levels of categorical explanatory variables (*taxa number*:*sub* *basin*, *taxa number*:*basin*, *taxa number*:*region*, *taxa number*:*Active long*‐*lived* DG, *taxa number*:*Passive* DG). The parameter estimates of GLMM curves, and their statistical tests were given in the relation to the reference level, which was defined as the first level of spatial extent (reach extent) and dispersal group (*Active short*‐*lived* DG) in our study. For the reference levels, the *z*‐tests determined whether the estimates were significantly greater than zero. For the other levels, *z*‐tests checked the significant differences in the strength of relationship between Akaike weights and the taxa number (i.e. differences in the estimated slopes), as well as differences in Akaike weights for a given number of taxa (i.e. differences in the estimated intercepts) between each level of the categorical variables and the reference level. We summed the estimated slope and intercept values of each level of categorial explanatory variables and the reference level (Cum. *est*.) to obtain the predicted slopes and y‐axis intercepts of GLMM curves separately for each spatial extent and dispersal group.

**TABLE E3:** The empirical Akaike weights of OFD patterns and the evidence ratios of OFD pairs were compared to the same statistical parameters predicted by GLMM models for all mean taxa numbers obtained through empirical observations (see in Table 3).

Taxa number	*Active long*‐*lived* DG						*Active short*‐*lived* DG						*Passive* DG					
	Bimodal		Unimodal		BI/UNI		Bimodal		Unimodal		BI/UNI		Bimodal		Unimodal		BI/UNI	
	*Emp*	*Pred*	*Emp*	*Pred*	*Emp*	*Pred*	*Emp*	*Pred*	*Emp*	*Pred*	*Emp*	*Pred*	*Emp*	*Pred*	*Emp*	*Pred*	*Emp*	*Pred*
12.07	0.67	0.6	0.18	0.34	3.82	1.76	**–**	0.86	**–**	0.14	**–**	6.14	**–**	0.49	**–**	0.52	**–**	0.94
12.81	0.52	0.57	0.43	0.38	1.21	1.50	**–**	0.85	**–**	0.15	**–**	5.67	**–**	0.46	**–**	0.55	**–**	0.84
13.45	0.53	0.54	0.43	0.41	1.22	1.32	**–**	0.84	**–**	0.16	**–**	5.25	**–**	0.43	**–**	0.58	**–**	0.74
14.21	0.46	0.51	0.53	0.45	0.88	1.13	**–**	0.82	**–**	0.18	**–**	4.56	**–**	0.4	**–**	0.61	**–**	0.66
14.78	**–**	0.48	**–**	0.49	**–**	0.98	**–**	0.81	**–**	0.19	**–**	4.26	0.44	0.37	0.56	0.64	0.79	0.58
16.83	**–**	0.4	**–**	0.6	**–**	0.67	**–**	0.76	**–**	0.24	**–**	3.17	0.26	0.29	0.74	0.72	0.36	0.40
17.76	**–**	0.36	**–**	0.65	**–**	0.55	**–**	0.73	**–**	0.26	**–**	2.81	0.19	0.26	0.81	0.75	0.23	0.35
20.42	**–**	0.26	**–**	0.77	**–**	0.34	**–**	0.65	**–**	0.34	**–**	1.91	0.07	0.18	0.93	0.83	0.08	0.22
24.23	**–**	0.15	**–**	0.89	**–**	0.17	0.62	0.52	0.33	0.47	1.85	1.11	**–**	0.1	**–**	0.9	**–**	0.11
28.12	**–**	0.08	**–**	0.95	**–**	0.08	0.28	0.38	0.71	0.61	0.40	0.62	**–**	0.05	**–**	0.95	**–**	0.05
30.35	**–**	0.06	**–**	0.97	**–**	0.06	0.27	0.31	0.73	0.68	0.37	0.46	**–**	0.04	**–**	0.96	**–**	0.04
33.49	**–**	0.03	**–**	0.99	**–**	0.03	0.11	0.22	0.89	0.77	0.12	0.29	**–**	0.02	**–**	0.98	**–**	0.02

*Note*: The table presented the empirical mean taxa number obtained through empirical observations (*Taxa number*), the empirical mean Akaike weight of bimodal and unimodal OFDs (*bimodal emp*, *unimodal emp*), the empirical evidence ratios for bimodal‐unimodal OFD pairs (*BI/UNI emp*), the predicted Akaike weights of bimodal and unimodal OFDs (*bimodal pred*, *unimodal pred*), and the predicted evidence ratios for bimodal–unimodal OFD pairs (*BI/UNI pred*) given by GLMM models. The data were given separately for each dispersal group.

## References

[ece311663-bib-0001] Anderson, D. R. , Burnham, K. P. , & Thompson, W. L. (2000). Null hypothesis testing: Problems, prevalence, and an alternative. The Journal of Wildlife Management, 64(4), 912–923. 10.2307/3803199

[ece311663-bib-0002] Angeler, D. G. , & Allen, C. R. (2016). Quantifying resilience. Journal of Applied Ecology, 53, 617–624. 10.1111/1365-2664.12649

[ece311663-bib-0003] Aspin, T. W. H. , Khamis, K. , Matthews, T. J. , Milner, A. M. , O'Callaghan, M. J. , Trimmer, M. , Woodward, G. , & Ledger, M. E. (2019). Extreme drought pushes stream invertebrate communities over functional thresholds. Global Change Biology, 25(1), 230–244. 10.1111/gcb.14495 30346098 PMC7379955

[ece311663-bib-0004] Barton, K. (2022). MuMIn: Multi‐model inference . R Package Version 1.47.1. https://CRAN.R‐project.org/package=MuMIn

[ece311663-bib-0005] Bêche, L. A. , McElravy, E. P. , & Resh, V. H. (2006). Long‐term seasonal variation in the biological traits of benthic‐macroinvertebrates in two Mediterranean‐climate streams in California, USA. Freshwater Biology, 51(1), 56–75. 10.1111/j.1365-2427.2005.01473.x

[ece311663-bib-0006] Borhidi, A. (2003). Magyarország növénytársulásai (p. 610). Akadémiai Kiadó.

[ece311663-bib-0007] Brooks, M. E. , Kristensen, K. , van Benthem, K. J. , Magnusson, A. , Berg, C. W. , Nielsen, A. , Skaug, H. J. , Maechler, M. , & Bolker, B. M. (2017). glmmTMB: Balances speed and flexibility among packages for zero‐inflated generalized linear mixed modeling. The R Journal, 9(2), 378–400. 10.32614/RJ-2017-066

[ece311663-bib-0008] Brown, B. L. , Swan, C. M. , Auerbach, D. A. , Campbell Grant, E. H. , Hitt, N. P. , Maloney, K. O. , & Patrick, C. (2011). Metacommunity theory as a multispecies, multiscale framework for studying the influence of river network structure on riverine communities and ecosystems. Journal of the North American Benthological Society, 30(1), 310–327. 10.1899/10-129.1

[ece311663-bib-0009] Brown, J. H. (1984). On the relationship between abundance and distribution of species. The American Naturalist, 124(2), 255–279. 10.1086/284267

[ece311663-bib-0010] Bruno, D. , Gutiérrez‐Cánovas, C. , Sánchez‐Fernández, D. , Velasco, J. , & Nilsson, C. (2016). Impacts of environmental filters on functional redundancy in riparian vegetation. Journal of Applied Ecology, 53(3), 846–855. 10.1111/1365-2664.12619

[ece311663-bib-0011] Burnham, K. P. , & Anderson, D. R. (2002). Model selection and multimodel inference: A practical information‐theoretic approach. Springer‐Verlag. 10.1007/b97636

[ece311663-bib-0012] Burnham, K. P. , Anderson, D. R. , & Huyvaert, K. P. (2011). AIC model selection and multimodel inference in behavioral ecology: Some background, observations, and comparisons. Behavioral Ecology and Sociobiology, 65(1), 23–35. 10.1007/s00265-010-1029-6

[ece311663-bib-0013] Collins, S. L. , & Glenn, S. M. (1991). Importance of spatial and temporal dynamics in species regional abundance and distribution. Ecology, 72(2), 654–664. 10.2307/2937205

[ece311663-bib-0014] Collins, S. L. , & Glenn, S. M. (1997). Effects of organismal and distance scaling on analysis of species distribution and abundance. Ecological Applications, 7(2), 543–551. 10.2307/2269519

[ece311663-bib-0015] De Cáceres, M. , & Legendre, P. (2009). Associations between species and groups of sites: Indices and statistical inference. Ecology, 90(12), 3566–3574. 10.1890/08-1823.1 20120823

[ece311663-bib-0016] De Cáceres, M. , Legendre, P. , & Moretti, M. (2010). Improving indicator species analysis by combining groups of sites. Oikos, 119(10), 1674–1684. 10.1111/j.1600-0706.2010.18334.x

[ece311663-bib-0017] de Vries, A. , & Ripley, B. D. (2022). ggdendro: Create dendrograms and tree diagrams using 'ggplot2' . R Package Version 0.1.23. https://CRAN.R‐project.org/package=ggdendro

[ece311663-bib-0018] Death, R. G. (2010). Disturbance and riverine benthic communities: What has it contributed to general ecological theory? River Research and Applications, 26(1), 15–25. 10.1002/rra.1302

[ece311663-bib-0019] Dodds, W. K. , Gido, K. , Whiles, M. R. , Fritz, K. M. , & Matthews, W. J. (2004). Life on the edge: The ecology of Great Plains prairie streams. BioScience, 54(3), 205–216. 10.1641/0006-3568(2004)054[0205:LOTETE]2.0.CO;2

[ece311663-bib-0020] Elzhov, T. V. , Mullen, K. M. , Spiess, A.‐N. , & Maintainer, B. B. (2022). minpack.l m: R interface to the Levenberg‐Marquardt nonlinear least‐squares algorithm found in MINPACK, plus support for bounds. R Package Version 1.2‐2. https://CRAN.R‐project.org/package=minpack.lm

[ece311663-bib-0021] Gafta, D. , Stoica, I. , & Coldea, G. (2021). The ranked species occupancy curves reflect the dominant process of species sorting: Evidence from forest/scrub communities. Journal of Vegetation Science, 32, e13086. 10.1111/jvs.13086

[ece311663-bib-0022] Garamszegi, L. Z. , & Mundry, R. (2014). Multimodel‐inference in comparative analyses. In L. Garamszegi (Ed.), Modern Phylogenetic Comparative Methods and Their Application in Evolutionary Biology. Springer. 10.1007/978-3-662-43550-2_12

[ece311663-bib-0023] Gaston, K. J. , & Lawton, J. H. (1989). Insect herbivores on bracken do not support the core‐satellite hypothesis. American Naturalist, 134(5), 761–777. 10.1086/285010

[ece311663-bib-0024] Hanski, I. (1982). Dynamics of regional distribution: The core and satellite species hypothesis. Oikos, 38(2), 210–221. 10.2307/3544021

[ece311663-bib-0025] Heatherly, T. , Whiles, M. R. , Gibson, D. J. , Collins, S. L. , Huryn, A. D. , Jackson, J. K. , & Palmer, M. A. (2007). Stream insect occupancy‐frequency patterns and metapopulation structure. Oecologia, 151(2), 313–321. 10.1007/s00442-006-0596-8 17091283

[ece311663-bib-0026] Heino, J. (2008). Temporally stable abundance–occupancy relationships and occupancy frequency patterns in stream insects. Oecologia, 157(2), 337–347. 10.1007/s00442-008-1078-y 18553109

[ece311663-bib-0027] Heino, J. (2015). Deconstructing occupancy frequency distributions in stream insects: Effects of body size and niche characteristics in different geographical regions. Ecological Entomology, 40(5), 491–499. 10.1111/een.12214

[ece311663-bib-0028] Hering, D. , Buffagni, A. , Moog, O. , Sandin, L. , Sommerhäuser, M. , Stubauer, I. , Feld, C. , Johnson, R. , Pinto, P. , Skoulikidis, N. , Verdonschot, P. , & Zahrádková, S. (2003). The development of a system to assess the ecological quality of streams based on macroinvertebrates – Design of the sampling programme within the AQEM project. International Review of Hydrobiology, 88(3–4), 345–361. 10.1002/iroh.200390030

[ece311663-bib-0029] Hering, D. , Moog, O. , Sandin, L. , & Verdonschot, P. F. M. (2004). Overview and application of the AQEM assessment system. Hydrobiologia, 516, 1–20. 10.1023/B:HYDR.0000025255.70009.a5

[ece311663-bib-0030] Hui, C. (2012). Scale effect and bimodality in the frequency distribution of species occupancy. Community Ecology, 13(1), 30–35. 10.1556/ComEc.13.2012.1.4

[ece311663-bib-0031] Hui, C. , & McGeoch, M. A. (2007). Modeling species distributions by breaking the assumption of self‐similarity. Oikos, 116(12), 2097–2107. 10.1111/j.2007.0030-1299.16149.x

[ece311663-bib-0032] Jenkins, D. G. (2011). Ranked species occupancy curves reveal common patterns among diverse metacommunities. Global Ecology and Biogeography, 20(3), 486–497. 10.1111/j.1466-8238.2010.00617.x

[ece311663-bib-0033] Johnson, J. B. , & Omland, K. S. (2004). Model selection in ecology and evolution. Trends in Ecology & Evolution, 19(2), 101–108. 10.1016/j.tree.2003.10.013 16701236

[ece311663-bib-0034] Kammer, P. M. , & Vonlanthen, C. M. (2009). The shape of occupancy distributions in plant communities: The importance of artefactual effects. Web Ecology, 9(1), 8–23. 10.5194/we-9-8-2009

[ece311663-bib-0035] Kassambara, A. , & Mundt, F. (2020). factoextra: Extract and visualize the results of multivariate data analyses. Package Version 1.0.7, 1(3). https://CRAN.R‐project.org/package=factoextra

[ece311663-bib-0036] Korkeamäki, E. , Elo, M. , Sahlén, G. , Salmela, J. , & Suhonen, J. (2018). Regional variations in occupancy frequency distribution patterns between odonate assemblages in Fennoscandia. Ecosphere, 9(4), e02192. 10.1002/ecs2.2192

[ece311663-bib-0037] Leibold, M. A. , Holyoak, M. , Mouquet, N. , Amarasekare, P. , Chase, J. M. , Hoopes, M. F. , Holt, R. D. , Shurin, J. B. , Law, R. , Tilman, D. , Loreau, M. , & Gonzalez, A. (2004). The metacommunity concept: A framework for multi‐scale community ecology. Ecology Letters, 7(7), 601–613. 10.1111/j.1461-0248.2004.00608.x

[ece311663-bib-0038] Lüdecke, D. (2018). Ggeffects: Tidy data frames of marginal effects from regression models. Journal of Open Source Software, 3(26), 772. 10.21105/joss.00772

[ece311663-bib-0039] Malmqvist, B. , Zhang, Y. , & Adler, P. H. (1999). Diversity, distribution and larval habitats of north Swedish blackflies (Diptera: Simuliidae). Freshwater Biology, 42(2), 301–314. 10.1046/j.1365-2427.1999.444497.x

[ece311663-bib-0040] McGeoch, M. A. , & Gaston, K. J. (2002). Occupancy frequency distributions: Patterns, artefacts and mechanisms. Biological Reviews, 77(3), 311–331. 10.1017/S1464793101005887 12227519

[ece311663-bib-0041] Mehranvar, L. , & Jackson, D. A. (2001). History and taxonomy: Their roles in the core‐satellite hypothesis. Oecologia, 127(1), 131–142. 10.1007/s004420000574 28547164

[ece311663-bib-0042] Nee, S. , Gregory, R. D. , & May, R. M. (1991). Core and satellite species: Theory and artefacts. Oikos, 62(1), 83–87. 10.2307/3545451

[ece311663-bib-0043] Oksanen, J. , Gavin, S. , Blanchet, F. , Roeland, K. , Pierre, L. , Peter, M. , O'Hara, R. B. , Solymos, P. , Stevens, M. , Szoecs, E. , Wagner, H. , Matt, B. , Michael, B. , Ben, B. , Daniel, B. , Gustavo, C. , Michael, C. , Miquel, D. C. , Sebastien, D. , & James, W. (2022). vegan: Community Ecology Package. R Package Version 2.6‐4. https://CRAN.R‐project.org/package=vegan

[ece311663-bib-0044] Pedersen, T. (2023). patchwork: The composer of plots. R Package Version 1.1.3. https://CRAN.R‐project.org/package=patchwork

[ece311663-bib-0045] R Core Team . (2022). R: A language and environment for statistical computing. R Foundation for Statistical Computing.

[ece311663-bib-0046] Reice, S. R. , Wissmar, R. C. , & Naiman, R. J. (1990). Disturbance regimes, resilience, and recovery of animal communities and habitats in lotic ecosystems. Environmental Management, 14, 647–659. 10.1007/BF02394715

[ece311663-bib-0047] Renner, S. , Dalzochio, M. S. , Périco, E. , Sahlén, G. , & Suhonen, J. (2020). Odonate species occupancy frequency distribution and abundance–occupancy relationship patterns in temporal and permanent water bodies in a subtropical area. Ecology and Evolution, 10(14), 7525–7536. 10.1002/ece3.6478 32760546 PMC7391549

[ece311663-bib-0048] Sarremejane, R. , Cid, N. , Stubbington, R. , Datry, T. , Alp, M. , Cañedo‐Argüelles, M. , Cordero‐Rivera, A. , Csabai, Z. , Gutiérrez‐Cánovas, C. , Heino, J. , Forcellini, M. , Millán, A. , Paillex, A. , Pařil, P. , Polášek, M. , Tierno de Figueroa, J. M. , Usseglio‐Polatera, P. , Zamora‐Muñoz, C. , & Bonada, N. (2020). DISPERSE, a trait database to assess the dispersal potential of European aquatic macroinvertebrates. Scientific Data, 7(1), 386. 10.1038/s41597-020-00732-7 33177529 PMC7658241

[ece311663-bib-0049] Sarremejane, R. , Stubbington, R. , England, J. , Sefton, C. E. M. , Eastman, M. , Parry, S. , & Ruhi, A. (2021). Drought effects on invertebrate metapopulation dynamics and quasi‐extinction risk in an intermittent river network. Global Change Biology, 27, 4024–4039. 10.1111/gcb.15720 34032337

[ece311663-bib-0050] Schmera, D. , Heino, J. , & Podani, J. (2022). Characterising functional strategies and trait space of freshwater macroinvertebrates. Scientific Reports, 12, 12283. 10.1038/s41598-022-16472-0 35854038 PMC9296484

[ece311663-bib-0051] Suhonen, J. (2021). Spatial and temporal changes in occupancy frequency distribution patterns of freshwater macrophytes in Finland. Ecology and Evolution, 11(14), 9553–9562. 10.1002/ece3.7773 34306642 PMC8293721

[ece311663-bib-0052] Suhonen, J. , & Jokimäki, J. (2019). Temporally stable species occupancy frequency distribution and abundance‐occupancy relationship patterns in urban wintering bird assemblages. Frontiers in Ecology and Evolution, 7, 129. 10.3389/fevo.2019.00129

[ece311663-bib-0053] Suhonen, J. , Paasivirta, L. , Rantala, M. J. , Jukka, S. , & Suutari, E. (2022). Macroinvertebrate species occupancy frequency distribution patterns in eutrophic lakes. Aquatic Ecology, 56(1), 201–212. 10.1007/s10452-021-09909-7

[ece311663-bib-0054] Symonds, M. R. E. , & Moussalli, A. (2011). A brief guide to model selection, multimodel inference and model averaging in behavioural ecology using Akaike's information criterion. Behavioral Ecology and Sociobiology, 65(1), 13–21. 10.1007/s00265-010-1037-6

[ece311663-bib-0055] Tokeshi, M. (1992). Dynamics of distribution in animal communities: Theory and analysis. Researches on Population Ecology, 34(2), 249–273. 10.1007/BF02514796

[ece311663-bib-0056] Tonkin, J. D. , Altermatt, F. , Finn, S. , Heino, J. , Olden, J. D. , Pauls, S. U. , & Lytle, D. A. (2018a). The role of dispersal in river network metacommunities: Patterns, processes, and pathways. Freshwater Biology, 63, 141–163. 10.1111/fwb.13037

[ece311663-bib-0057] Tonkin, J. D. , Heino, J. , & Altermatt, F. (2018b). Metacommunities in river networks: The importance of network structure and connectivity on patterns and processes. Freshwater Biology, 63, 1–5. 10.1111/fwb.13045

[ece311663-bib-0059] Van Looy, K. , Tonkin, J. D. , Floury, M. , Leigh, C. , Soininen, J. , Larsen, S. , Heino, J. , LeRoy Poff, N. , Delong, M. , Jähnig, S. C. , Datry, T. , Bonada, N. , Rosebery, J. , Jamoneau, A. , Ormerod, S. J. , Collier, K. J. , & Wolter, C. (2019). The three Rs of river ecosystem resilience: Resources, recruitment, and refugia. River Research and Applications, 35, 107–120. 10.1002/rra.3396

[ece311663-bib-0060] van Rensburg, B. J. , McGeoch, M. A. , Matthews, W. , Chown, S. L. , & Van Jaarsveld, A. S. (2000). Testing generalities in the shape of patch occupancy frequency distributions. Ecology, 81(11), 3163–3177. 10.2307/177408

[ece311663-bib-0061] Verberk, W. C. E. P. , Siepel, H. , & Esselink, H. (2008). Life‐history strategies in freshwater macroinvertebrates. Freshwater Biology, 53(9), 1722–1738. 10.1111/j.1365-2427.2008.02035.x

[ece311663-bib-0062] Verberk, W. C. E. P. , van der Velde, G. , & Esselink, H. (2010). Explaining abundance‐occupancy relationships in specialists and generalists: A case study on aquatic macroinvertebrates in standing waters. Journal of Animal Ecology, 79(3), 589–601. 10.1111/j.1365-2656.2010.01660.x 20202007

[ece311663-bib-0063] Verberk, W. C. E. P. , Van Noordwijk, C. G. E. , & Hildrew, A. G. (2013). Delivering on a promise: Integrating species traits to transform descriptive community ecology into a predictive science. Freshwater Science, 32(2), 531–547. 10.1899/12-092.1

[ece311663-bib-0064] Wagenmakers, E. J. , & Farrell, S. (2004). AIC model selection using Akaike weights. In. Psychonomic Bulletin and Review, 11(1), 192–196. 10.3758/BF03206482 15117008

[ece311663-bib-0065] Wickham, H. (2016). ggplot2: Elegant graphics for data analysis. Springer.

[ece311663-bib-0066] Wilkes, M. A. , Edwards, F. , Jones, J. I. , Murphy, J. F. , England, J. , Friberg, N. , Hering, D. , Poff, N. L. R. , Usseglio‐Polatera, P. , Verberk, W. C. E. P. , Webb, J. , & Brown, L. E. (2020). Trait‐based ecology at large scales: Assessing functional trait correlations, phylogenetic constraints and spatial variability using open data. Global Change Biology, 26(12), 7255–7267. 10.1111/gcb.15344 32896934

